# Applying biomarkers as paleoenvironmental indicators to reveal the organic matter enrichment of shale during deep energy exploration: a review

**DOI:** 10.1039/d3ra04435a

**Published:** 2023-08-29

**Authors:** Abiodun Busuyi Ogbesejana, Bo Liu, Shuo Gao, Segun Ajayi Akinyemi, Oluwasesan Michael Bello, Yu Song

**Affiliations:** a State Key Laboratory of Shale Oil and Gas Enrichment Mechanism and Effective Development Beijing 100101 China liubo@nepu.edu.cn; b Institute of Unconventional Oil & Gas, Northeast Petroleum University Daqing 163318 China; c Department of Applied Chemistry, Federal University Dutsin-Ma P. M. B. 5001, Dutsin-Ma Katsina State Nigeria; d Department of Geology, Faculty of Science, Ekiti State University, Ado-Ekiti P. M. B. 5363 Ado-Ekiti Ekiti State Nigeria; e Key Laboratory of Tectonics and Petroleum Resources (China University of Geosciences), Ministry of Education Wuhan 430074 China

## Abstract

Analysis of biomarkers in geological materials such as shales is very crucial because they can provide useful information on the depositional conditions and environments, organic matter input, thermal maturity as well as the geological age of shales in some cases. The paleoenvironment, and its impact on organic matter enrichment of the shales, plays a vital role in the exploration and development of the resource. Paleoenvironmental reconstruction can be conducted using elemental, isotopic, maceral, and biomarker proxies. However, the literature on the biomarkers for paleoenvironment reconstruction to reveal the organic matter enrichment of shales in many petroleum systems throughout the world is still insufficient. Hence, this paper seeks to critically review the application of biomarkers during paleoenvironmental reconstruction in shales. The uses of biomarkers as indicators of modern and ancient marine and brackish/saline lacustrine depositional environments are considered. This review shows that biomarkers could be used to establish the sedimentary depositional environments, redox conditions, and organic matter enrichments of shales that are critical to deep energy exploitation. Nevertheless, despite the fact that biomarkers are significant indicators of depositional conditions, secondary processes such as source facies, thermal maturity, migration, and reservoir alteration can greatly influence their uses as paleoenvironmental condition indicators in source rocks and oils. Hence, for a reliable paleoenvironmental evaluation, there is a need to combine isotopic, elemental and maceral proxies with biomarkers.

## Abstract 2

This review shows that biomarkers could be used to establish the sedimentary depositional environments, redox conditions, and organic matter enrichments of shales that are critical to the deep energy exploitation.

## Graphical abstract

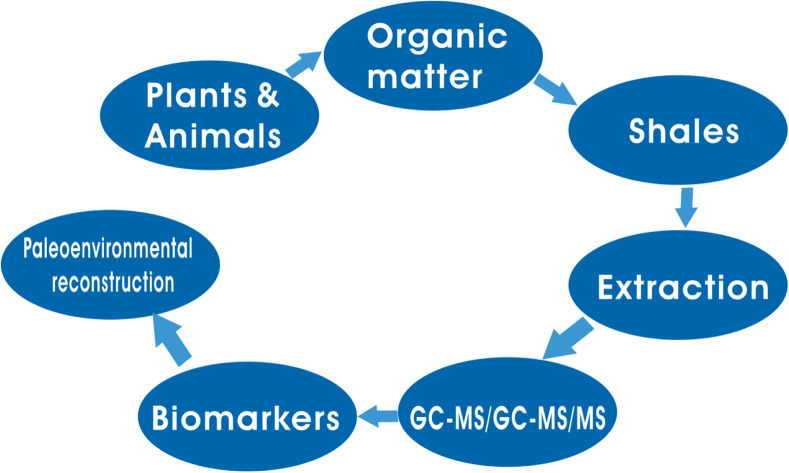

## 1 Introduction

Paleoenvironmental reconstruction has gained global attention of researchers because of its importance to conventional hydrocarbon resources and unconventional oil and gas exploration. Different depositional environments and conditions may have different assemblages of organisms, and thus contribute different biomarkers to the sediment. For instance, terrigenous, marine, deltaic, and hypersaline environments all show characteristic differences in biomarker compositions.^1–3^ The redox state of the environment in which shales were deposited can be inferred from the abundance and ratios of certain biomarkers in the shales.^3^ Biomarkers are complex molecular fossils that have been preserved in sediments from once-living organisms.^1–3^ A biomarker is a substance that maintains the structure of its biological precursor.^1,3,4^ The use of biomarkers to infer paleoflora, paleoenvironments, and the origin of life on Earth, as well as to provide a zonation for diagenetic change, has been reported.^1,4–7^ Early research has shown that the information contained in biomarker distribution can be successfully used for the differentiation and assessment of depositional environments,^8–10^ particularly for the characterization and distinction of ancient marine and non-marine petroleum source rocks,^11,12^ or even more detailed sub-environments, such as lacustrine freshwater and hypersaline, marine carbonate and deltaic sediments.^12–16^ The components of sediment extracts and oils are a reflection of both paleoenvironmental conditions and precursor compounds in the organisms that contributed organic matter (OM) at the time of sediment deposition, and thus can provide valuable information about the organic matter input and the prevailing depositional environment.^8,9^ The source facies can be distinguished by comparing structurally similar chemicals in sediments and crude oils with their likely biological precursors.^8,9,17,18^ The thermal history of the oil can be reconstructed by altering the biomarker structure.^3,17,18^ Some biomarkers are biochemical processes or environmental indicators. Triterpenoids and steroids were thoroughly investigated. During diagenesis, sterols are easily changed, whereas polycyclic terpenoids are more resistant^19^ Any organic structures that emerge in sediments before the oil is expelled from the source rock can be used for correlation, not just biological structures, for example, diamondoids were formed during catagenesis.^20^ As a result, biomarkers from oil provide valuable information on depositional paleoenvironments,^21^ maturity,^22^ and organic input, as well as, in some instances, the age of the source rocks.^23,24^ Numerous studies on paleoenvironments based on marine and lacustrine sediments have been conducted.^25–32^ Depositional conditions of the organic-rich source rocks have long been a main focus of research into the vast petroleum-prone source rock formation.^32–35^

**Fig. 1:**
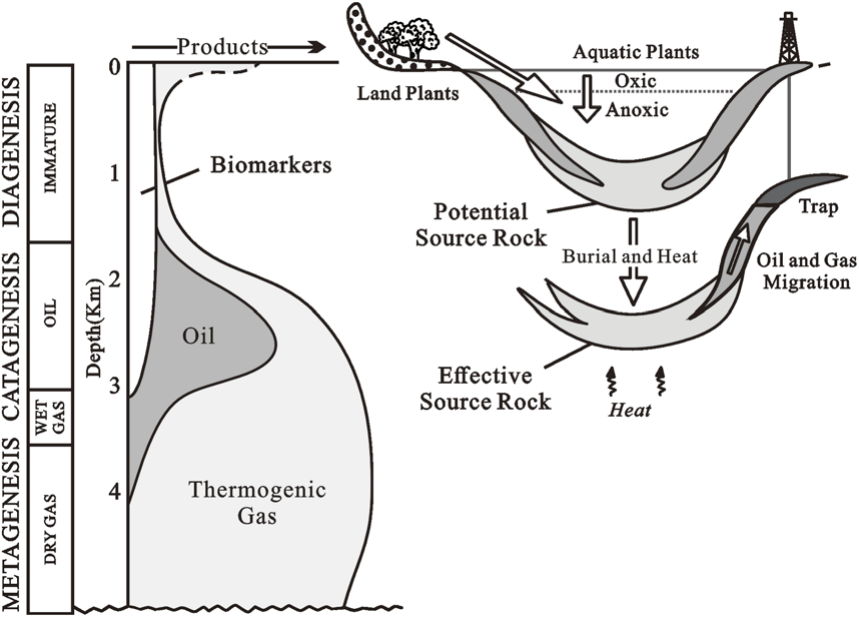
Generalized evolution of organic matter after deposition. Many biomarkers transform to other structures during late diagenesis and much of catagenesis before their destruction during late catagenesis and metagenesis (left).^3^

The essential structures of biomarkers are mainly preserved during sedimentation and diagenesis (Fig. 1).^3,6^ The term “diagenesis” refers to the biological, physical, and chemical changes that occur in organic matter in sediments before major heat (usually 50 °C)-induced alterations.^1,3,4,6^ Catagenesis is the thermal alteration of organic materials in rocks caused by burial and heating at temperatures between 50 and 150 °C under typical burial settings over millions of years.^1,3,4,6^ Biomarkers undergo structural changes during catagenesis that can be used to determine the degree of heating of their source rocks or the amount of oil expelled from these rocks.^3,6,36,37^ Furthermore, because biomarkers indicate a distinct group of contributing organisms, their distribution in an effective source rock serves as a fingerprint that can be utilized to link the rock to the expelled crude oil that may have traveled hundreds of kilometers.^3,6,36,37^ Before greenschist metamorphism, organic molecules are broken to gas at temperatures between 150 and 200 °C, a process known as metagenesis. Because of their volatility, biomarkers lose a significant amount of concentration or are eliminated in these settings through several paths: (1) cracking of very mature particulate organic matter, (2) breakdown of residual oil in petroleum source rocks, and (3) secondary cracking of oil in reservoir rocks can all result in deep hydrocarbon gas accumulations.^5,7,38^ In the oil–oil, and oil–source rock correlation studies, biomarkers are commonly utilized.^36,37^*n*-Alkane indices, in combination with sterane and aromatic ratios, can help distinguish between terrestrial and marine organic matter inputs; the ratio of hopanes to steranes can help distinguish prokaryotic *versus* eukaryotic input; and various saturated and aromatic hydrocarbon ratios can help suggest thermal maturity, lithology, and depositional environments.^19^

Shale is defined as a fine-grained sedimentary rock that exhibits fissility and a more general classification of mudstone.^39^ Organic-rich shale refers to the shale with elevated total organic carbon contents (generally TOC >2.0% (ref. 40)), which commonly acts as important petroleum sources, reservoirs for shale oil and gas, and seals in conventional reservoirs.^41–46^ Organic-rich shales are widespread in salinized lacustrine basins throughout China, such as the Middle Permian Lucaogou Formation in Junggar and Santanghu basins in northwest China,^47,48^ the Upper Jurassic Yanchang Formation in Ordos Basin in central China,^49^ the Upper Cretaceous Qingshankou and Nenjiang formations in Songliao Basin in northeast China,^50^ and the Eocene Shahejie Formation in Bohai Bay Basin in east China.^43,51^ These salinized lacustrine organic-rich shale (SLORS) have been proven as crucial source rocks for conventional oil, as well as sources and reservoirs for unconventional oil.^52^ The abundance of organic carbon and heteroatoms in shale layers, which are deposited in source rocks in the form of organic matter, leads to the generation of hydrocarbon through thermal maturation. The solid-state organic compound (known as kerogen) of these atoms breaks down and undergoes a significant structural and compositional transformation during thermal maturation.^53,54^

To date, however, while there are many studies reported on the paleoenvironmental reconstruction in shales using elemental, maceral, and isotopic proxies, the literature on the biomarkers for paleoenvironment reconstruction to reveal the organic enrichment of shales in many petroleum systems throughout the world is still insufficient. Thus, the aim of this paper is to critically review the significance of biomarkers during paleoenvironmental reconstruction in shales.

## 2 Lacustrine *versus* marine environments

Lake and ocean environments differ in many ways, resulting in different quantities and qualities of OM in their sediments.^3,6,55–58^ Due to the smaller size and shallower depth, lakes generally receive a more turbulent influx. Similarly, compared to the deep oceans, land-derived nutrients are more abundant in lakes, boosting primary production.^3,6,55–58^ Lake sedimentation rates (∼1 m/1000 years) frequently exceed those in seas and oceans (∼1–10 cm/1000 years), therefore OM gets buried more quickly, aiding preservation.^3,6,55–58^ Because they receive huge amounts of terrigenous OM and deposited quite quickly (10–100 cm/1000 years), coastal marine sediments are more similar to lake sediments.^3,6,55–58^ Lacustrine sediments typically contain tens of percent TOC, whereas deep ocean sediments contain only a few tenths. Lacustrine benthic creature diversity is lower, and their bioturbation depth is lower than that of marine fauna.^58^ In seawater, dissolved sulfate is a major ion, whereas it is usually low or absent in lakes.^59^ As a result, sulfate reduction is significant in the microbial reworking of marine OM, but not in the reworking of lacustrine OM. Carroll and Bohacs (2001) suggested a three-fold lithofacies categorization for lacustrine petroleum source rocks that accounts for the most relevant characteristics.^58^ These lithofacies correspond to their algal-terrestrial, algal, and hypersaline algal organic facies, and comprise the fluvial-lacustrine, fluctuating pro-fundal, and evaporative lithofacies. All three lacustrine facies are found in the Eocene Green River Formation in Wyoming and the Upper Permian non-marine facies of the southern Junggar Basin in China.^3,6^

**Fig. 2:**
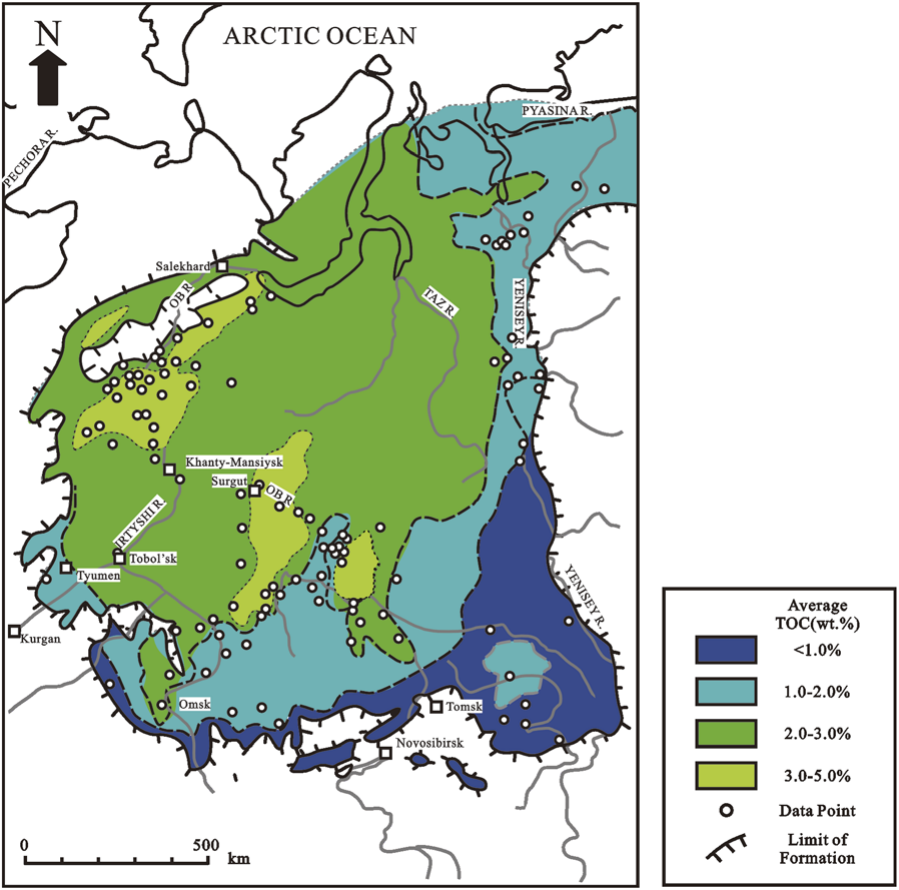
Map of total organic carbon (TOC) distribution for Lower-Middle Jurassic shales, including the Middle Jurassic Tyumen Formation in West Siberia.^60^

**Fig. 3:**
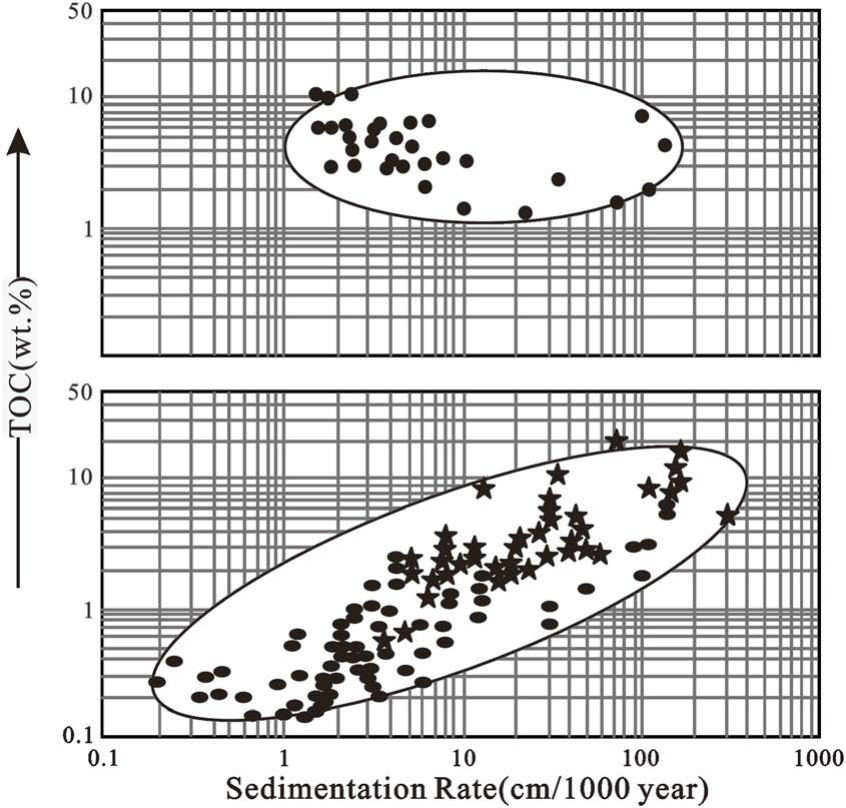
Schematic comparison of sedimentation rate *versus* total organic carbon in sediments.^66–69^

OM has a varied pattern of increasing concentration further from the source of the organic matter at the margin of the basin toward deep water in deltaic or paralic environments dominated by terrigenous detritus.^59^ The Mississippi and Mahakam deltas are typically modern examples, whereas the Miocene Mahakam Delta and the Lower-Middle Jurassic in West Siberia are typically ancient examples (Fig. 2). Although most deltaic environments are very oxic, OM can also be preserved if it is buried quickly, successfully protecting it from metazoan attack (Fig. 3).

## 3 Oxic *versus* anoxic depositional condition

Biomarker compositions can be used to distinguish oils from various source rocks, but they can also be used to show regional differences in organic facies within the same source rock or between oils from the same source rock.^61^ Because biomarker patterns in oils are inherited from their source rocks, these uses are possible. Within the same source rock, lateral and vertical variations of organic facies emerge from differences in the type of organic matter and the characteristics of the depositional environment. An organic facies is a mappable subdivision of a stratigraphic unit defined by its organic components.^62–65^ Understanding the paleo-oceanographic influences on petroleum source rock deposition can help with regional mapping of organic facies and predicting promising regions for future development.^62,64,65^ Under certain atmospheric circumstances, aerobic microorganisms rapidly oxidize organic materials from dead plants or animals. Aqueous sedimentation of organic matter can occur under a variety of redox circumstances, with the availability of molecular oxygen being the most important factor. The terms in Table 1 can be used to characterize the various redox conditions and their corresponding microbial biofacies (determined by metabolism).

**Table 1:** Common terminology describing the redox conditions in sedimentary environments and the metabolism of the corresponding microbial populations (biofacies)^3,64^

Depositional environment	Microbial biofacies	Oxygen content (ml l^−1^)	
		Tyson and Pearson^70^	Demaison and More^35^
Oxic	Aerobic	>1	2–8
Dysoxic	Dysaerobic	0.1–1	0.2–2
Suboxic	Quasi-anaerobic	0.1–1	0–0.2
Anoxic	Anaerobic	0	0

### 3.1 Oxic condition

**Fig. 4:**
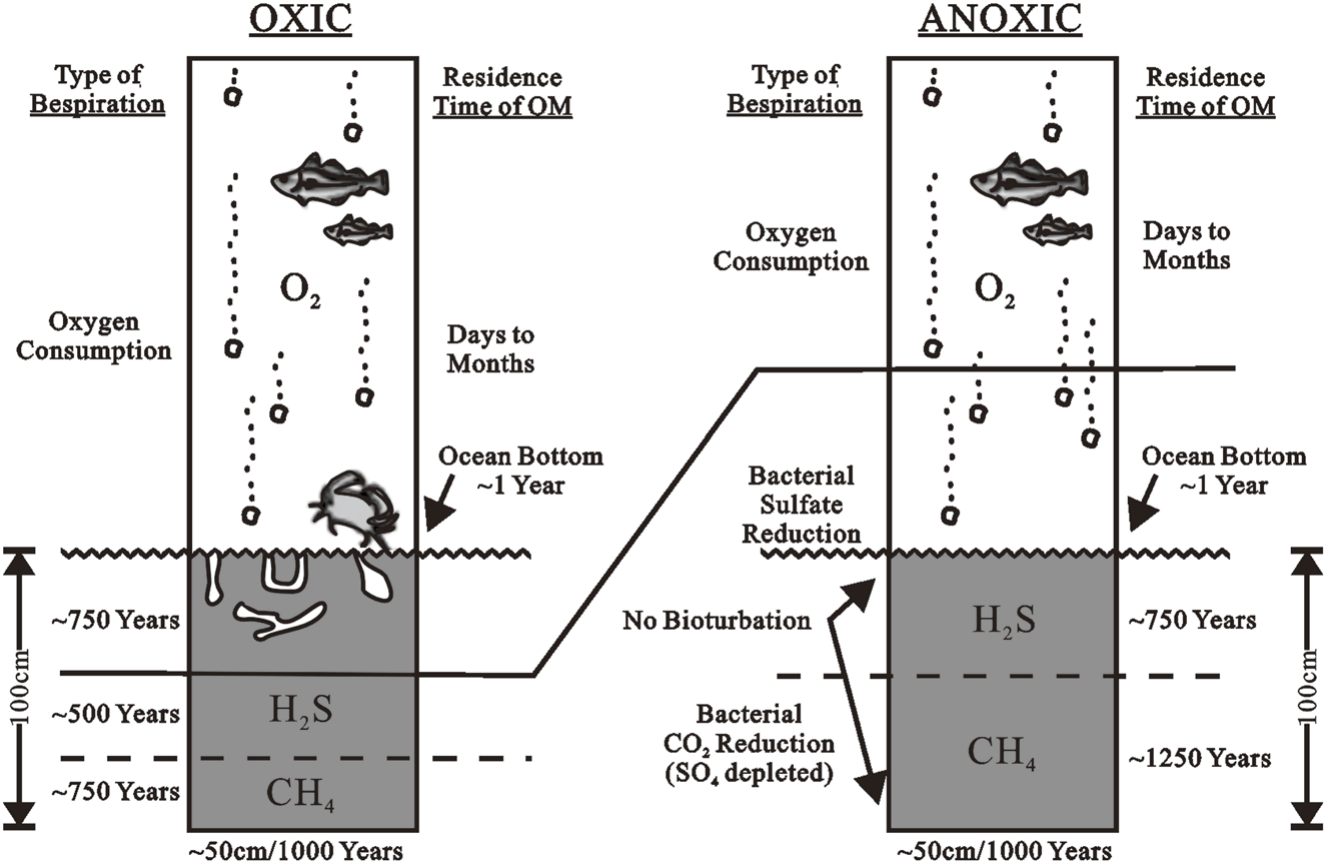
The O_2_–H_2_S–CH_4_ level in oxic (left) and anoxic (right) depositional environments generally result in poor and good preservation of deposited organic matter, respectively.^71^

**Fig. 5:**
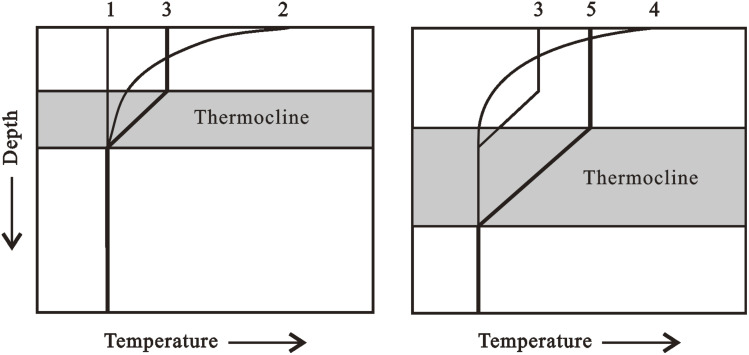
Schematic showing the formation of a thermocline in watermass. If air temperature rises above water temperature for water having constant temperature *versus* depth (1), then the surface water begins to warm (2). Mixing of surface waters by wind lowers surface temperature, resulting in the formation of an isothermal layer having a higher temperature than the thermocline and deeper water (3, left). Further warming (4) and mixing of the surface water result in net downward transport of heat and a deeper, and commonly thicker, thermocline (5).^3^

Aerobic bacteria and other organisms decompose organic materials settling from the photic zone in an oxic deposition (Fig. 4). There are 6–8 ml of oxygen per liter in normal seawater. Biological oxygen demand (BOD) is created by respiratory processes. If enough organic matter remains after all available oxygen has been used up, anaerobic organisms continue to oxidize it with other oxidants like nitrate or sulfate. In the water column or the bottom sediments, the boundary between aerobic and anaerobic metabolism (oxic *versus* anoxic environments) can occur. Metazoa, such as multicellular burrowing organisms like clams and worms, typically bioturbated benthic sediments that contain interstitial oxygen. Massive textures without lamination characterize oxic deposits preserved in the geologic record. Because of the combined effects of BOD and restricted replenishment by oxygenated water, lakes and oceanic basins can become oxygen-depleted (stagnant). Basin shape, water temperature, and salinity gradients are all elements that influence water recharge. For example, a thermocline is a layer of water in which the temperature decreases with depth more than the underlying and above water (Fig. 5). When the air temperature is high and the shallow water gets more heat than it loses through radiation and convection, thermoclines can form in lakes and the ocean. When the surface water heats, a small negative temperature gradient occurs. Although wind mixing of the surface waters lowers the surface temperature, the overall impact is downward heat transmission and the establishment of an isothermal layer warmer than the underlying water. As a result, a strong thermocline forms between the isothermal surface layer and the cooler water beneath it. Warm surface waters in the oceans usually extend to a depth of 150–300 m. The thickness of the underlying thermocline ranges from 300 to 900 m. Below the thermocline, the temperature drops more slowly and reaches 1–4 °C towards the bottom of the ocean.

Because of the humid atmosphere and lack of major seasonal temperature variations, equatorial lakes, such as Lake Tanganyika in the East African rift-lake system, are particularly prone to severe thermoclines.^71^ Due to the thermocline, oxygen lost from deep water due to BOD is not rapidly replaced by mixing with shallower water, and anoxia develops. Anoxic conditions exist below 150 meters in Lake Tanganyika, which is 1500 meters deep. The Eocene Green River Formation in Colorado and Utah have laminated, organic-rich marlstones that were deposited in a vast anoxic lake. Non-marine Lower Cretaceous source rocks from China (Songliao Basin), Brazil (Lagoa Feia Formation), and West Africa (Bucomazi Formation) are also instances.

A halocline or density-stratified water column can form when fresh water is pumped into a silled marine basin with limited evaporation, especially when deep saline water is separated from open ocean water by the sill. The Black Sea is primarily a sluggish marine basin. Excess fresh water from riverine input pours into the Mediterranean Sea through a 27 m deep sill at the Bosphorus. The upshot of this positive water balance is low-salinity surface water relative to the Black Sea's deeper, more saline water and a permanent halocline at depth. The halocline is a chemocline that distinguishes between oxic and anoxic conditions. At 80–100 m depth, the current chemocline is found within the photic zone, where hydrogen sulfide first develops and oxygen vanishes. Isorenieratene and related compounds found in Black Sea sediments suggest that photosynthetic green sulfur bacteria (Chlorobiaceae) have been active in the Black Sea for at least 6000 years and that anaerobic water penetration of the photic zone is not a recent phenomenon.^69^ The Upper Jurassic of West Siberia (Bazhenov Formation) and the North Sea (Kimmeridge hot shales), as well as the latest Albian in North America, are examples of anoxic marine source rocks deposited under similar conditions (Mowry Shale).

### 3.2 Anoxic conditions

**Fig. 6:**
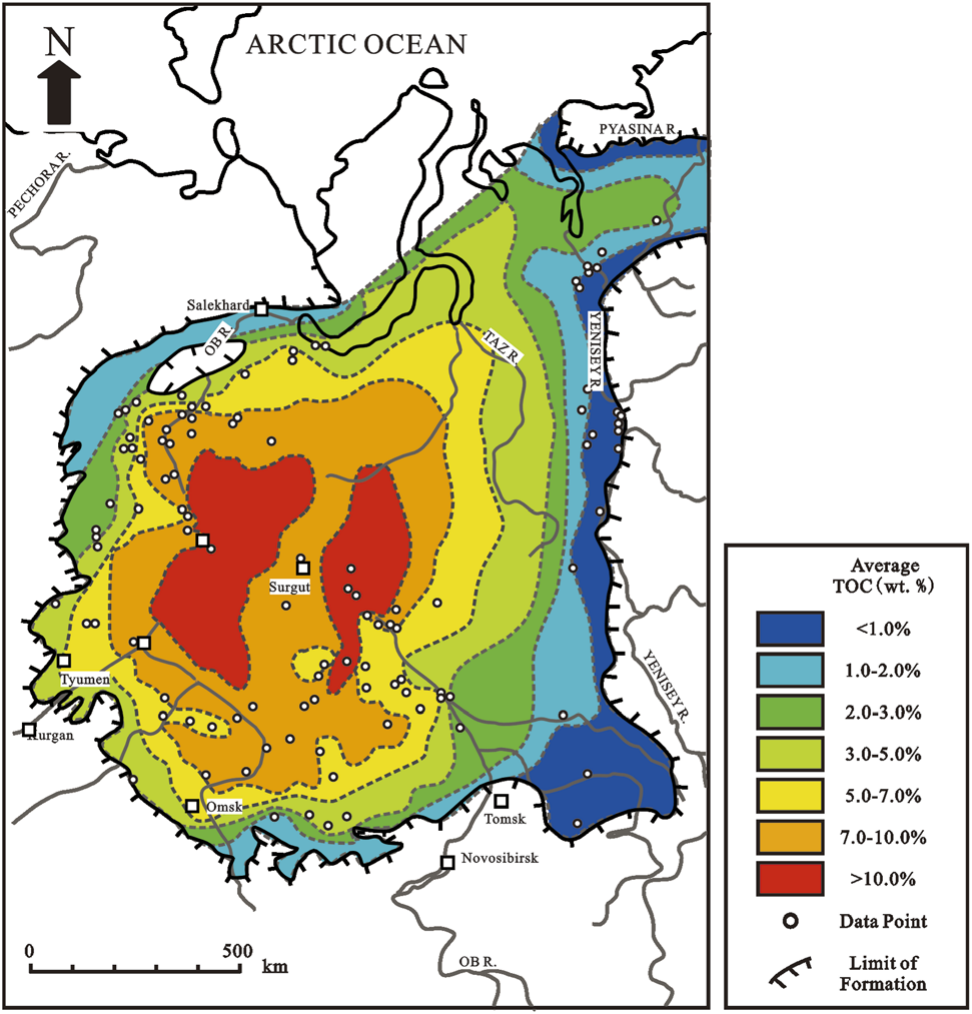
Map of total organic carbon (TOC) distribution for marine shales of the Upper Jurassic Bazhenov Formation in West Siberia, deposited in a large anoxic silled basin.^85^

OM and fine-grained sediments get concentrated in concentric or bull's-eye patterns in deep quiet water near source-rock depocenters in anoxic marine silled basins and anoxic lakes where sediment thickness is the greatest.^72^ The Black Sea and the Caspian Sea are modern instances of this concentric distribution of organic carbon, while the Upper Jurassic in West Siberia (Fig. 6), the lower Jurassic of the northern North Sea, and the lower Toarcian and Hettangian/Sinemurian of the Paris Basin are ancient examples. Anoxic conditions range from less than ∼1 wt% total organic carbon to more than 20 wt% total organic carbon (TOC). Turbidity currents and related gravity flows may muddle the aforementioned concentric distributions by transporting organic matter or sediments into deep water *via* pathways that aren't predicted by basic depositional models.

The implications of oxic *vs.* anoxic deposition on the quantity rather than the quality of preserved OM were examined by Pedersen and Calvert.^72^ Anoxic conditions appear to enhance the preservation of hydrogen-rich, oil-prone organic materials. Peters and Simoneit (1982),^73^ for example, found identical TOC content in alternating layered (anoxic) and homogeneous (oxic) diatomaceous oozes in the Gulf of California, similar to.^74^ Higher Rock-Eval pyrolysis hydrogen indices and lower oxygen indices imply that the laminated zones in these sediments contain more hydrogen-rich organic matter than the homogeneous zones. If anoxia is unimportant in the preservation of OM, it is difficult to explain the widespread correlation between oil-prone, organic-rich petroleum source rocks and faunal or sedimentologic traits indicating anoxia, according to.^75^ The majority of source rocks are laminated and lack signs of active infauna. Biomarkers and supporting parameters in petroleum source rock extracts indicate anoxic conditions (*e.g.*, high vanadium/nickel porphyrin, low pristane/phytane, and high C_35_ homohopane indices).^3^

Because both metazoa (multicellular aerobic organisms) and aerobic bacteria demand higher amounts of oxygen, aerobic breakdown of organic matter is significantly limited in anoxic or suboxic water (less than ∼0.2 ml oxygen/l water) (Fig. 4, right). Bioturbation of benthic sediments does not occur below ∼0.1 ml of oxygen/l water due to the absence of metazoa, leaving only anaerobic bacteria and probably some benthic foraminifera to rework the organic matter. Interstitial oxygen, nitrate, Mn^4+^ oxides, Fe^3+^ oxides, and sulfate are the oxidants employed by benthic organisms in general.^76,77^ Because sulfate is abundant in seawater (0.028 M), sulfate reduction is usually the major mode of respiration after the advent of anaerobic conditions in marine settings.^78^ The absence of bioturbation allows the formation of tiny laminations that record depositional cycles, as seen in effective petroleum source rocks. Glacial varves in fjords, for example, reflect yearly depositional cycles that often comprise a thin layer of dark-colored organic-rich clay grading upward from a layer of light-colored sand or silt. Laminated sediments at British Columbia's Saanich Inlet, a silted, fjord-like area, contain up to 9% organic matter.^79^ Free hydrogen sulfide (H_2_S) generated by sulfate-reducing bacteria is accumulated in euxinic sediments under marine, anoxic conditions.^80^

During sediment deposition, traces of animal activity provide information about water chemistry.^81^ For example, few ichnofossils (trace fossils) other than very shallow Helminthoides burrows consisting of short, monospecific horizontal mining and grazing traces can be found in Lower to Middle Triassic mudrocks from the Barents Sea.^75^ Due to the lack of evidence for burrowing, these mudrocks were deposited in dysoxic to anoxic conditions, resulting in better OM preservation, high TOC, and high algal/amorphous OM. In marine sediments, Savrda (1995) summarized connections between oxicity and burrow type or density.^81^ However, systematic correlations between redox conditions and bioturbation in lacustrine sediments are poorly established.^82^ Anaerobic degradation of organic materials is less effective thermodynamically than aerobic degradation.^83^ This finding backs up the widely held assumption that anoxia is to blame for the increased preservation of hydrogen- and lipid-rich organic materials in petroleum source rocks.^55^ Even when organic productivity is high, and oxic conditions present, organic matter is generally lost by sedimentation and diagenesis. Most polar locations in current oceans, for example, have high primary productivity, but low organic carbon in the oxic bottom sediments. Organic productivity, rather than anoxia, is the fundamental restriction on the buildup of organic-rich marine sediments, according to.^60,73^ They cited sources that suggested that the rates of the breakdown of organic matter under oxic and anoxic circumstances are equal and cannot be used to imply increased retention of OM under anoxic conditions, based mostly on laboratory incubation tests. They found no increase in carbon content in alternating anoxic and oxic sediments in the central Gulf of California.^74^ In comparison to analogous, oxygenated environments, data from anoxic sediments in the Black Sea reveal that organic carbon accumulation rates are not especially high.^84^ The distribution of current organic-rich sediments around the planet does not appear to be correlated with high productivity in the underlying water column.^55^ Because of the significant circulation of cold, oxygen-rich waters that successfully satisfy all BOD imposed by settling the organic matter, surface waters near Antarctica, for example, display great productivity, but the underlying sediments are organic-lean. Modern organic-rich sediments are found in areas with high productivity and anoxia at the bottom of the water column.^3^

## 4 Methods of biomarker analyses

The analytical methods applied in the analyses of biomarkers and characterizations are reported below.

### 4.1 Extraction

The shale samples are usually crushed with agate mortar and powdered to less than 100 mesh size before extraction. About 50 g powdered samples (although this generally depends on the extractable organic matter (EOM) contents in shale) are Soxhlet extracted with an azeotropic mixture of dichloromethane : methanol (93 : 7, v/v) for 72 h. To eliminate elemental sulfur from the extracts, activated copper powder is widely used. The excess solvent is then distilled out with a rotary evaporator to a 3 ml aliquot volume. After transferring the aliquot into a clean, weighed vial with a micropipette, the remaining solvent is removed under nitrogen gas flow at a temperature below 50 °C.^86^ Alternatively, microwave-assisted extraction (MAE) can be applied using hexane/acetone (1 : 1). Other solvents that can be used for the MAE include tetrachloroethylene, methylene chloride/acetone (1 : *l*), toluene/methanol (l0 : *l*), methylene chloride, and toluene/methanol (1 : 10). Soxtec extraction can also be employed using hexane/acetone (*l* : *l*), and that for ultra-sonic extraction, methylene chloride/acetone (9 : 1) is used.^87^

### 4.2 Column chromatography

To fractionate extracts, column chromatography (some automatic instruments are also used to separate the EOM into SARA) with silica gel/alumina as the stationary phase is commonly utilized. A standard glass column measures 50 cm in length and has a 0.5 cm internal diameter. DCM and light petroleum spirits are used to rinse the column twice (petroleum ether). The column is then rinsed with *n*-hexane and plugged with a cotton wool to serve as a resting pad for the stationary phase, which is silica gel (SiO_2_). The stationary phase (SiO_2_) is then added. Two (2 g) of alumina (Al_2_O_3_) is used to stabilize the surface. The saturated, aromatic hydrocarbon and polar fractions are eluted using 70 ml of *n*-hexane, 70 ml of dichloromethane/*n*-hexane (2 : 1, v/v), and 70 ml of dichloromethane/methanol (1 : 1, v/v). Each fraction is recovered by carefully evaporating solvents on a rotary evaporator, followed by the removal of the remaining solvent under the influence of a nitrogen gas stream.^87^ Gas chromatography (GC), gas chromatography-mass spectrometry (GC-MS), and gas chromatography coupled to tandem mass spectrometry (GC/MS/MS) can now be used to analyze the recovered saturated hydrocarbon, aromatic hydrocarbon, and polar fractions.^88^

### 4.3 Gas chromatography (GC)

The aliphatic fractions are commonly analyzed by capillary gas chromatography for the characterization of the n-alkanes, pristane, and phytane (GC). Typically, a Hewlett Packard 5890 Series II is used, which is equipped with a Gerstel on-column injector, an electronic pressure control (EPC), a fused silica capillary column (HP Ultra I) of 50 m length, 0.2 mm inner diameter, and 0.33 m film thickness, as well as a standard flame ionization detector (FID). At a flow rate of 1 ml min^−1^, hydrogen is widely utilized as a carrier gas (pressure controlled). The oven temperature is commonly programmed from 90 °C (hold time 5 min) to 310 °C at a rate of 4 °C min^−1^ (for some unusual biomarkers, some special temperature programs are also used). A Multichrom 2-online data system (Fisons) is commonly used to store and process retention times and peak areas.

### 4.4 Gas chromatography-mass spectrometry (GC-MS)

**Fig. 7:**
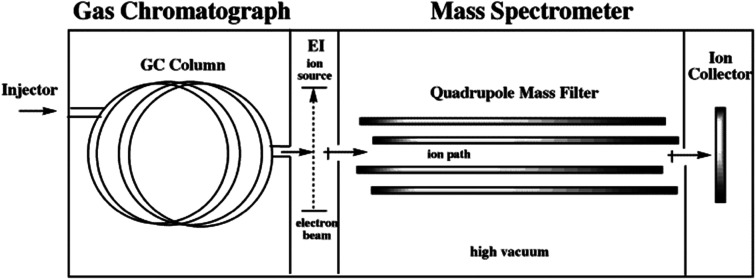
Schematic diagram of GC-MS.

Gas chromatography-mass spectrometry (GC-MS) (Fig. 7) analyses are exceedingly sensitive, allowing for the analysis of very small amounts of material. The combination of MS and GC for detection offers a unique capability for identifying unknown substances or confirming the presence of target molecules in complex mixtures.^89–92^ GC-MS is a widely used analytical technique for a variety of purposes, including the analysis of geological samples, pharmaceuticals, pesticides, environmental pollutants, xenobiotics, and toxins.^93–95^ During the separation and detection of substances, GC-MS instrumentation necessitates the utilization of increased temperatures in the injector, column, detector, and transfer line to the MS.^89–92^ The injector temperature must be higher than the boiling point of the primary substance in the mixture to be examined. The standard capillary columns employed in these analyses have a column length of 15 to 30 m. The flow rate of the mobile phase (gas) is modest, around 1 ml min^−1^ or less. The current GC-MS technique has a significant restriction in that it can only analyze a limited number of volatile, thermally stable compounds. The individual biomarkers are commonly identified based on comparison of elution sequences, relative retention times, and mass spectra with the NIST Chemistry WebBook (https://webbook.nist.gov/chemistry/) and those previously reported.^96–98^

### 4.5 Gas chromatography-mass spectrometry-mass spectrometry (GC-MS-MS)

Gas chromatography tandem mass spectrometry (GC/MS/MS) is a more powerful analytical technique. The most common application of GC/MS/MS is for trace quantitative analysis in complex matrices, such as geological samples.^99,100^ The system's ability to choose against the matrix (lower chemical noise) is an important performance component to consider. The signal-to-noise ratio (S/N) can be used to demonstrate this.^99,100^ Furthermore, an S/N ratio ensures that instruments are not contaminated during installation, while low-level precision and instrument detection limits (IDL) provide the full image.^99,100^ The physical and chemical properties of analytes of interest, as well as their interaction with the analytical column's stationary phase, are used to separate samples in a gaseous form for GC-MS/MS analysis. The analytes enter the tandem mass spectrometer (MS/MS) after departing the analytical column, which is made up of two scanning mass analyzers separated by a collision cell.^99,100^ In the collision cell, fragments picked in the first analyzer react with inert gas, resulting in additional fragmentation. These daughter product ions are then resolved and analyzed in the third quadrupole. Liquids, gases, and solids can all be analyzed using GC-MS/MS. The sample is directly introduced into the GC for liquids. Gaseous components are transferred directly into the GC using gastight syringes. Solvent extraction, outgassing, or pyrolysis analysis are all options for solids analysis. Following that, the analytes of interest are measured by comparing them to external or internal standards. GC-MS/MS is highly suited for the detection of unknown volatile components using mass fragmentation patterns and mass transitions associated with the unknown analyte, in addition to quantification.

### 4.6 Comprehensive two-dimensional gas chromatography time-of-flight mass spectrometry (GC × GC-TOFMS)

**Fig. 8:**
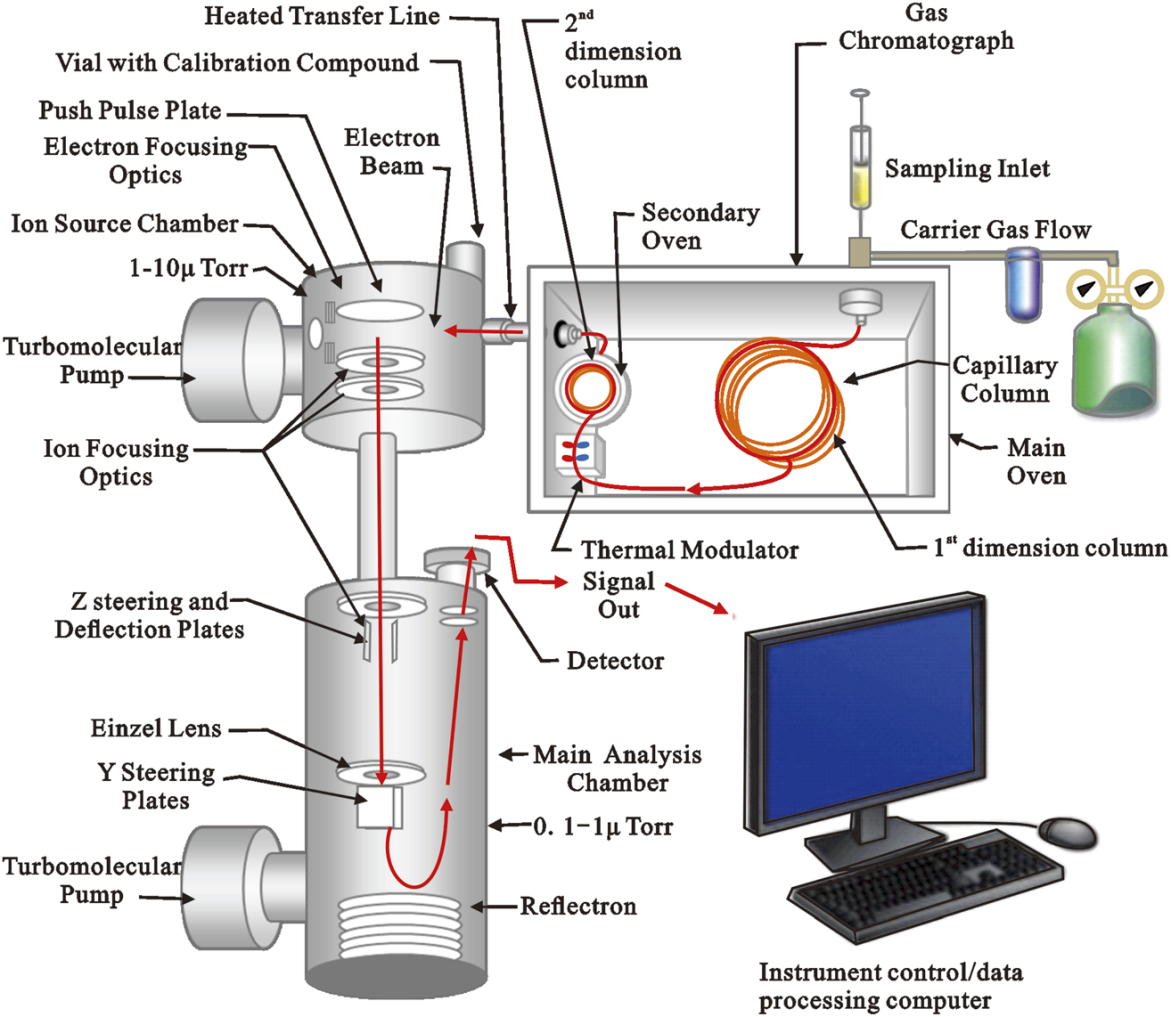
Schematic diagram of GC × GC-TOFMS-instrument.

Comprehensive two-dimensional gas chromatography (GC × GC) has shown remarkable potential for the investigation of complex mixtures since its inception in the 1990s.^101–103^ Furthermore, considering the importance of biomarkers in geochemical and environmental studies, a precise and complete investigation of their composition in oils and source rocks is required. Capillary gas chromatography (GC), which is frequently hyphenated with mass spectrometry (MS),^90–93^ and tandem MS (MS-MS) are the most popular techniques used to study biomarkers.^99,100^ Although the abilities of these techniques are well established, there are certain limitations. The fundamental constraints of both GC-MS and GC-MS-MS are the extremely complex mixture of crude oils and shales, as well as the poor resolution of GC. Oil mixtures are not properly clarified, despite the better separation capabilities of multiple reaction monitoring (MRM) or MS-MS techniques. Comprehensive two-dimensional gas chromatography (GC × GC) is one of the most powerful analytical methods for separating complex mixtures with great resolution. The GC × GC is great for separating complex matrices and identifying isomers and other molecules with similar chemical structures. In bidimensional space, different well-ordered chemical groups can be recognized, providing extra information on the molecular composition. For retrieving biomarker structural information encoded in complex petroleum samples, detection using a time of flight mass spectrometry (TOFMS) device is required after the GC × GC separations. Because mass spectra are available for chemical identification and structure elucidation, the TOFMS can be regarded as a third analytical dimension. The non-scanning capabilities of TOF technology, as well as its rapid acquisition rate, are two of its most important advantages. On the one hand, the non-scanning capability is the most significant advantage over scanning MS instruments (quadrupole, ion trap), which produce non-skewed peaks. The entire chromatogram is obtained with non-distorted mass spectra, allowing for rapid spectral deconvolution and a reliable comparison with commercially available MS libraries. Rapid acquisition, on the other hand, is required for accurately reconstructing the narrow peaks generated during GC × GC separation modulation. To enable chromatographic resolution, provide sufficient MS information per peak, and efficiently deconvolve the compounds that co-elute, a high acquisition frequency is required during the time unit. The GC × GC-TOFMS (Fig. 8) is ideal for analyzing complex samples, especially for identifying unusual compounds or potential biomarkers that are rarely detected in routine 1D GC or GC-MS analyses, due to its better chromatographic resolution, well ordered 2D structures, mass spectral information, and deconvolution. GC × GC-TOFMS has been used in a variety of applications during the last few decades, including oil analysis.^93,104,105^ Aguiar *et al.* (2010, 2011) used GC × GC to identify new compounds in crude oils and to evaluate Brazilian petroleum systems.^106,107^ Ventura *et al.* (2010) investigated reservoir compartmentalization using GC × GC fingerprinting of several oils.^103^ The same group compared the compositions of different oils a year later using multiway principal components analysis (MPCA) on data from GC × GC studies.^94^ Silva *et al.* (2011) offered a detailed biomarker examination of Colombian oils,^106^ while Eiserbeck *et al.* (2011) accurately measured significant biomarkers using baseline separation in a GC × GC analysis.^94^ Oliveira *et al.* (2012) used GC × GC to explore branched-cyclic hydrocarbon heterogeneity in crude oil samples from two basins and to describe aromatic steroids and hopanoids from marine and lacustrine crude oils.^36,37^ Eiserbeck *et al.* (2012) compared the differences and advantages of various chromatographic separation and detection techniques, such as GC-MS, GC × GC-FID, and GCGC-TOFMS, for biomarker identification.^108^ The resolution of GC × GC is superior to that of classic 1D GC approaches, according to their research. The GC × GC-TOFMS technique produces a high-resolution separation and complete mass spectra across the whole chromatogram. Silva *et al.* (2013) and Soares *et al.* (2013) used GC × GC-TOFMS to examine information on oil maturity and biodegradation.^109,110^ Many saturated and aromatic biomarkers in extra heavy gas oil were successfully.^100,111^ Recently, Kiepper *et al.* (2014) used GC × GC-TOFMS to characterize the biomarkers in the crude oils from the Cumuruxatiba and Espírito Santo basins, Brazil.^112^

## 5 Biomarkers for paleoenvironmental reconstructions

Biomarker distributions in the OM have been widely utilized to describe the paleoenvironmental conditions.^3,6,95,113–120^ Diverse depositional environments may have different assemblages of organisms, resulting in different biomarkers being contributed to the sediment. For example, biomarker compositions differ significantly in terrigenous, marine, deltaic, and hypersaline environments.^3^ Details of biomarkers for paleoenvironments based on *n*-alkanes, isoprenoids, terpanes, and sterane, as well as some aromatic biomarkers, will be discussed in the following sections.

### 5.1 Saturated compounds

#### 5.1.1 Normal and branched alkanes

Coal, sediments, and petroleum all contain *n*-alkanes (*m*/*z* 85).^121^ They provide information about the biological sources of OM.^6,121^ Due to their biosynthesis from fatty acids by enzymatic decarboxylation, organism usually have odd numbers of C-atoms.^121^ Waxes of higher plants are responsible for *n*-alkanes in the C_27_ to C_31_ range, which show a distinct odd over even predominance (OEP).^122^ Short-chain *n*-alkanes (C_15_ to C_19_) with an OEP are thought to originate from algae (marine environment),^2^ implying that aquatic OM has a large role in deposition.^3,114,115^ Ratios can be used to express the influence of various contributors on the distribution of *n*-alkanes. For example, the ATR_HC_ (aquatic/terrigenous ratio of hydrocarbons, Wilkes *et al.* (1999)) is a parameter used to determine the amounts of aquatic to terrigenous derived *n*-alkanes, or short-chain to long-chain *n*-alkanes:^123^1
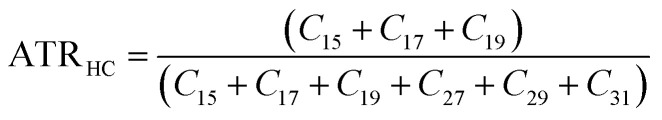


Long-chain *n*-alkanes, or organic matter derived from terrigenous OM, contribute significantly to values of 0.5. The ratio depends on the maturity of the samples and is only distinctive for immature organic matter. Samples of low maturity normally show the biogenic distribution of *n*-alkanes. With increasing organic matter maturity, the predominance of odd-numbered *n*-alkanes over even-numbered *n*-alkanes becomes less pronounced. Due to thermal cracking, an increase of short-chain *n*-alkanes can be observed, leading to equal proportions of odd and even-numbered *n*-alkanes.^121^ In estimating the thermal maturity of fossil fuels, the ratio of odd/even carbon-numbered *n*-alkanes has been used.^2,124^ These ratios are expressed as the carbon preference index, CPI^125^ or enhanced odd-to-even predominance, OEP.^124^ The CPI and OEP values less or greater than 1.0 indicate low thermal maturity while values around 1.0 suggest, but do not prove, that an oil or rock extract is thermally mature. The CPI or OEP values below 1.0 are unusual and typify low maturity oils or bitumen from carbonate or hypersaline environments.^3,126^ These ratios are affected by organic matter type and therefore are mostly applied with caution.2
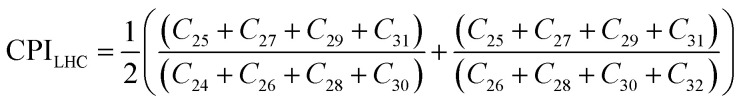


#### 5.1.2 Acyclic isoprenoids

**Fig. 9:**
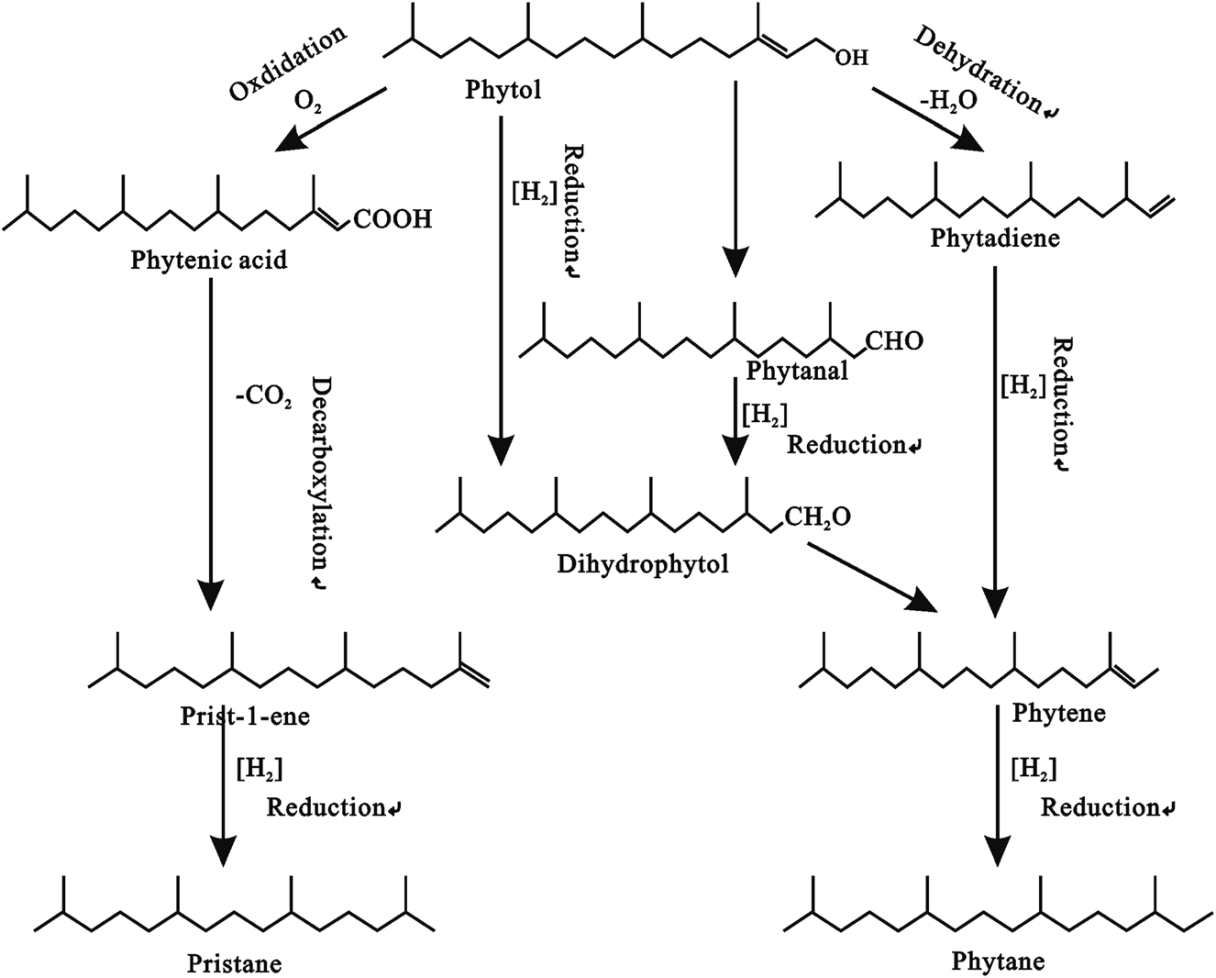
Formation of pristane and phytane from phytol.^140^

These compounds have been found in all types of geologic samples.^127^ They are known constituents of plants, animal tissues, and bacterial cell walls. The presence of acyclic isoprenoids in the biosphere is due to the phytol side chain of chlorophyll-a. They are composed of three types of linkages: head-to-tail (the most common, including compounds such as pristane (Pr), phytane (Ph), and homologs up to C_45_); tail-to-tail (squalane, perhydro-carotane, lycopane, and others); and head-to-head (C_32_–C_40_ typical of thermophilic and other archaebacteria). The phytol side chain of chlorophyll-a gives rise to Pr (2, 6, 10, 14-tetramethylpentadecane) (I) and Ph (2,6,10,14-tetramethylhexadecane) (II) (Fig. 9). The most abundant isoprenoids in the aliphatic hydrocarbon fraction are pristane and phytane.^6,128^ Phytane is the product of the dehydration and reduction of phytol, whereas pristane is derived from the oxidation and decarboxylation of phytol (Fig. 8). These isoprenoids are possible indicators of redox conditions during sedimentation and diagenesis because they represent the palaeoenvironmental conditions of source rocks.^129–131^ The pristane/phytane (Pr/Ph) ratio is a widely used geochemical parameter that has been utilized as a depositional environment indicator, but, it has low specificity due to thermal maturity interferences and preliminary assessment of OM inputs.^3^ It's also a common indicator of the redox conditions of the depositional environments. According to Ten Haven,^132^ high Pr/Ph (>3.0) indicates terrigenous input under oxic conditions, while low Pr/Ph (0.8) indicates anoxic, hypersaline, or carbonate environments. Low Pr/Ph levels (<2) suggest reducing conditions in aquatic depositional settings such as marine, fresh, and brackish water, whereas high values (up to 10) indicate oxidizing conditions in peat swamp depositional environments.^133^ Pr/Ph values are also influenced by maturity, and/or different precursors of pristane (tocopherols or chromas). Another important method for determining the source of organic matter is the Pr/*n*-C_17_ ratio. It is also widely used as an indicator of the redox potential of the depositional environment.

Squalane (III) has been proposed as a halophilic archaea indicator and hence a molecular diagnostic of hypersaline environment.^134,135^ Crocetane (IV) has been identified as a marker for methane-oxidizing archaea because it is depleted in ^13^C to a value as low as −100‰.^136^ Thiel *et al.* (2001) discovered that the *n*-C_23_ alkane, which is depleted in ^13^C (^13^C values less than −70), is an indicator of anaerobic methane oxidation.^137^ Methanogenic bacteria have been shown to contain isoprenoids such as penta- and tetramethylicosane.^138^ Archaea produce isoprenoids that are linked head-to-head.^139^

**Fig. 10:**
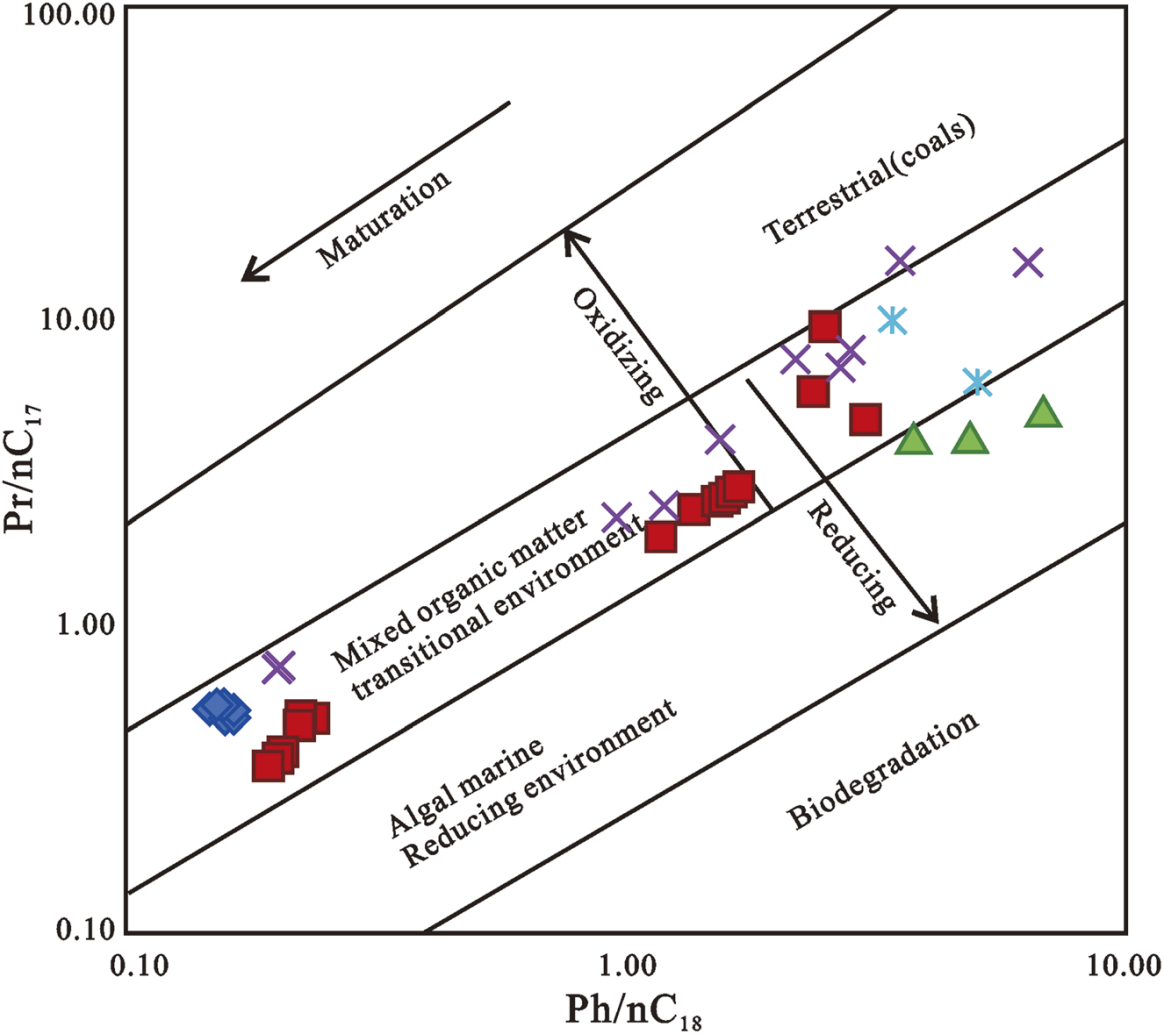
Plot of Pr/*n*-C_17_ against Ph/*n*-C_18_ ratios of oils from Niger Delta.^18,141^

OM derived from terrestrial sources is assigned to values above 0.6, whereas organic material generated from marine sources is attributed to values below 0.5.^6^ The pristane/normal alkane (Pr/*n*-C_17_) and phytane/normal alkane (Ph/*n*-C_18_) ratios have also been employed to determine redox conditions during sediment deposition (Fig. 10).^3,18,128,141^ The Pr/*n*-C_17_ and Ph/*n*-C_18_ ratios are also influenced by maturity and biodegradation.^141^

Botryococcane (V) is a branched hydrocarbon derived from botryococcene, an unsaturated hydrocarbon that has been linked to an organism that will only develop in a specific type of environment.^141^*Botryococcus braunii*, a fresh or brackish water alga, was shown to have botryococcane concentrations of 70 to 90 percent in its senescent phase. Moldowan and Seifert (1980) utilized the peculiar occurrence of this compound in *Botryococcus braunii* as evidence that certain oil deposits in Sumatra,^142^ Indonesia, were formed primarily from the prehistoric source material in a fresh or brackish lagoonal-type environment.^134^*B. braunii* can contribute both unsaturated hydrocarbons, which are potential precursors of botryococcanes, and long-chain *n*-alkanes to freshwater (lacustrine) sediments since it exists as two physiologically distinct clonal races.^142^ For the first time in an Australian crude oil, biomarkers found in coastal bitumens from the western Otway Basin confirmed the presence of substantial amounts of botryococcane.^143^ The waxy quality of the three main bitumen types found in the Otway Basin, as well as their botryococcane content, has been attributed to botryococcus blooms deposited in deep lakes under anoxic or micro-oxic circumstances.^144^

β-Carotane (VI), a saturated hydrocarbon generated from a pigment with a C_40_ carotenoid structure, was initially discovered in the Eocene Green River Shale of Colorado and has since been discovered in a variety of sedimentary rocks and crude oils. Although most carotenoids do not survive early diagenetic processes, β-carotane is well preserved in sediments and oils in many environments.^145–150^ β-Carotane is well known as a marker for saline and reducing lacustrine environments as well as extremely restricted marine environments.^11,151,152^ Carotenoids are the biological precursors of β-carotane and are produced by algae, cyanobacteria, and higher plants.^11,48,151,153^ β-Carotane is abundant in the eocene oils of the Bohai Bay Basin.^154^ The Eocene Shahejie (Es) formation, particularly the third (Es_3_) and fourth (Es_4_) members, is the principal source rock for both conventional and “shale oil-producing” formations in the Dongying depression, as several studies have shown.^155–157^ The levels of -carotane in the oils vary greatly between the Es_3_ and Es_4_ members. β-Carotane concentrations in the Es_4_ Member's oils ranged from 105 to 303 μg g^−1^ oil (averaged at 218 μg g^−1^ oil), whereas the Es_3_ Member's oils had a range of 73–145 μg g^−1^ oil. In a heavily biodegraded oil. The concentration of -carotane in a heavily biodegraded oil reached 1044 g g^−1^ oil. Wang *et al.* (2021) recently discovered β-carotane in a suite of lacustrine oils from the Dongying Depression, Bohai Bay Basin, Eastern China, and classified the oils into two groups based on β-carotane parameters.^158^ The ratios -carotane/C24 tetracyclic terpane, β-carotane/(C_19_ + C_20_) tricyclic terpanes, and β-carotane/(18(*H*)-22,29,30-trisnorneohopane+17(*H*)-22,29,30-trisnorhopane) have been proposed to be useful for distinguishing oils derived from different depositional environments.^158,159^

#### 5.1.3 Tricyclic and tetracyclic terpanes

The tricyclic terpanes (TT) (VII), which are abundant in source rock extracts and crude oils, form a pseudo-homologous series with carbon atoms spanning from C_19_ to C_54_. Because the higher members of the family are frequently hidden by hopanes in the *m*/*z* 191 mass chromatogram, they are generally recognized up to C_29_ compounds.^160^ Because the formation of C_22_TT and C_27_TT requires the cleavage of two carbon–carbon bonds, their abundance is particularly low.^3^ According to Ourisson *et al.* (1982), the tricyclic terpenes may have been derived from bacterial cell membranes.^161^ Furthermore, Aquino *et al.* (1983) claimed that a high abundance of tricyclic terpanes in crude oils could be linked to a higher contribution of marine algae.^162^ The relationship between high tricyclic terpane concentrations and “tasmanite” oil shale suggests that it may have originated from Tasmanites algae.^163–165^ However, the fact that it may be found in sediments and oils of various ages suggests that there must be other sources.^3^ In marine-sourced oils, the C_23_TT is frequently prominent, whereas a higher amount of C_19_TT and C_24_ tetracyclic terpane (TeT) usually indicates the presence of terrigenous organic.^13,162,166^ The ratio of C_26_TT to C_25_TT can be utilized to distinguish between marine and lacustrine source rocks.^3,167^ The relative distribution of tricyclic terpanes in combination with C_24_TeT has been widely used as a molecular parameter to distinguish terrestrial *versus* marine organic matter input, correlate crude oils and source rock extracts, predict source rock characteristics, and decipher source rock lithology.^3,13,167^ However, Samuel *et al.* (2010) recommended against such use because tricyclic terpanes and C_24_TeT distribution patterns in most oils are extremely similar globally.^168^

Tetracyclic terpanes (VIII) are found in a series ranging from C_24_ to C_27_. The ratio of C_24_TeT over C_26_TT is a source parameter due to differences in likely precursors of tetracyclic and tricyclic terpanes.^168–170^ Most marine oils formed from mudstones to carbonate source rocks contained abundant C_24_TeT.^171^ C_24_–C_27_ tetracyclic terpanes are often referred to as de-E-hopanes, or 17,21-secohopanes and are suggested to be more resistant against biodegradation and maturity effects than hopanes.^162^

**Fig. 11:**
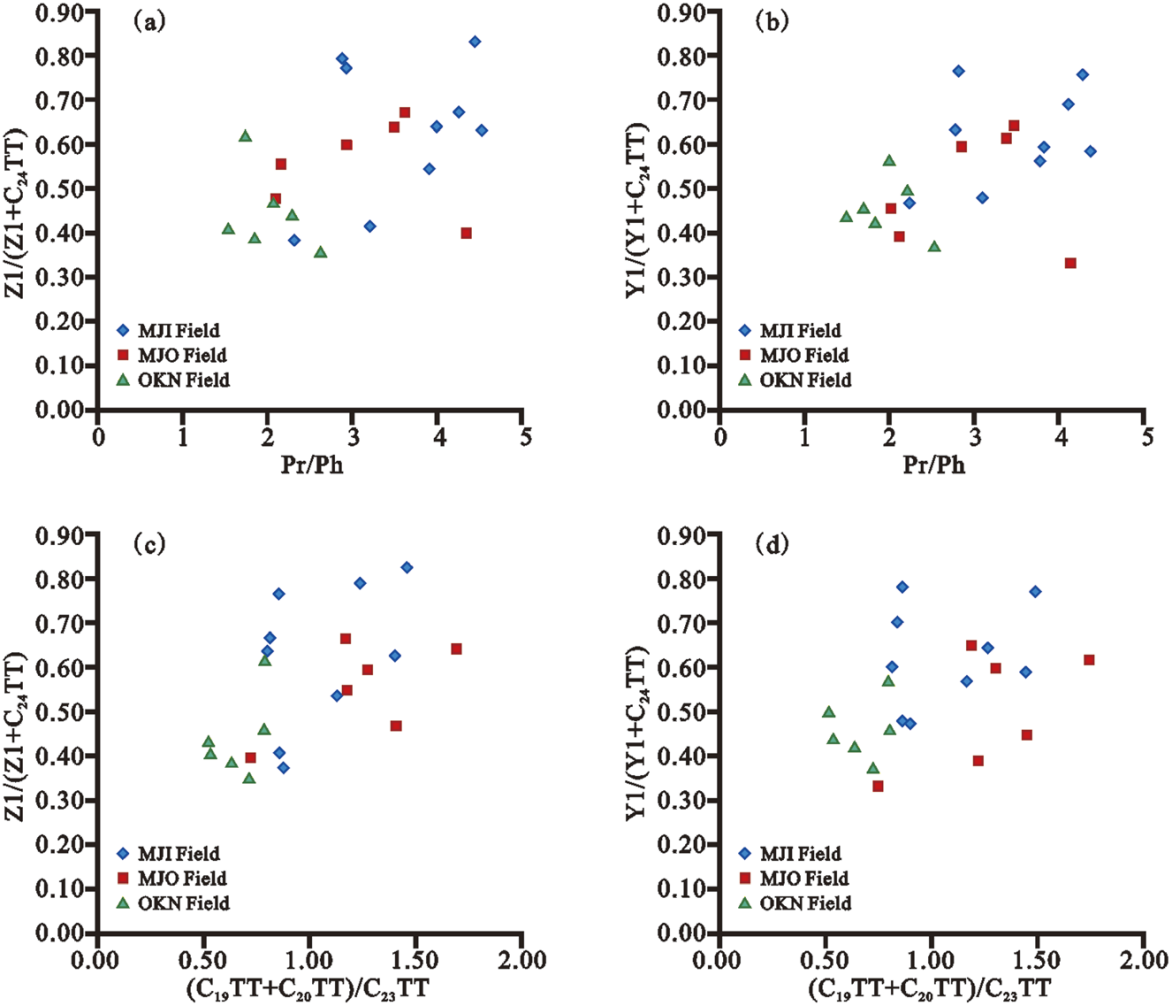
Cross plots of (a) Z1/(Z1 + C_24_TT) *vs.* Pr/Ph, (b) Y1/(Y1 + C_24_TT) *vs.* Pr/Ph, (c) Z1/(Z1 + C_24_TT) *vs.* (C_19_ + C_20_)TT/C_23_TT, and (d) Y1/(Y1 + C_24_TT) *vs.* (C_19_ + C_20_)TT/C_23_TT in source rock extracts from Offshore Niger Delta Basin, Nigeria.^169,170^

Unusual tri- and tetracyclic terpanes (C_21_ tricyclic terpanes, C_25_ tricyclic terpanes, C_27_ tetracyclic terpanes, C_24_-*des*-A-oleanane, C_24_-*des*-A-lupane, and C_24_-*des*-A-ursane) have recently been reported in crude oils and source rock extracts from the Pearl River Mouth Basin, the Beibuwan Basin, and the Liaohe Basin, China,^169,172^ and source rock extracts from Niger Delta Basin, Nigeria.^17^ According to Xiao *et al.* (2018), these uncommon compounds contain chemical structures that are comparable to oleanane, ursane, and lupane, and are thought to be derived from alcohols or ketone precursors found in higher plants.^169^ The higher abundance of these tri- and tetracyclic terpanes is likely due to the higher plant material input to the OM content of source rocks.^169^ Furthermore, the redox conditions and water depth in the depositional environment have a substantial impact on the distribution patterns of the compounds, and they may be easily generated under oxidizing conditions.^169^ Furthermore, Xiao *et al.* (2018) and Ogbesejana *et al.* (2020) found that the values of Pr/Ph in crude oils and source rock extracts from the Pearl River Mouth Basin, Beibuwan Basin, Liaohe Basin, China, and the Niger Delta Basin, Nigeria, were correlated with the values of Z1/(Z1 + C_24_TT) and Y1/(Y1 + C_24_TT). This means that Z1/(Z1 + C_24_TT), Y1/(Y1 + C_24_TT), and Pr/Ph are all constrained by the same depositional conditions, implying that these newly discovered tetracyclic terpanes were formed in both oxidative and reducing environments.^169,170^ The Z1/(Z1 + C_24_TT), Y1/(Y1 + C_24_TT), and (C_19_ + C_20_)TT/C_23_TT ratios are related to the Pr/Ph and (C_19_ + C_20_)TT/C_23_TT ratios, as shown in Fig. 11a–d.^169,170^ As a result, these compounds are derived from source rocks containing a mixture of higher plant and marine sources.^169^

**Fig. 12:**
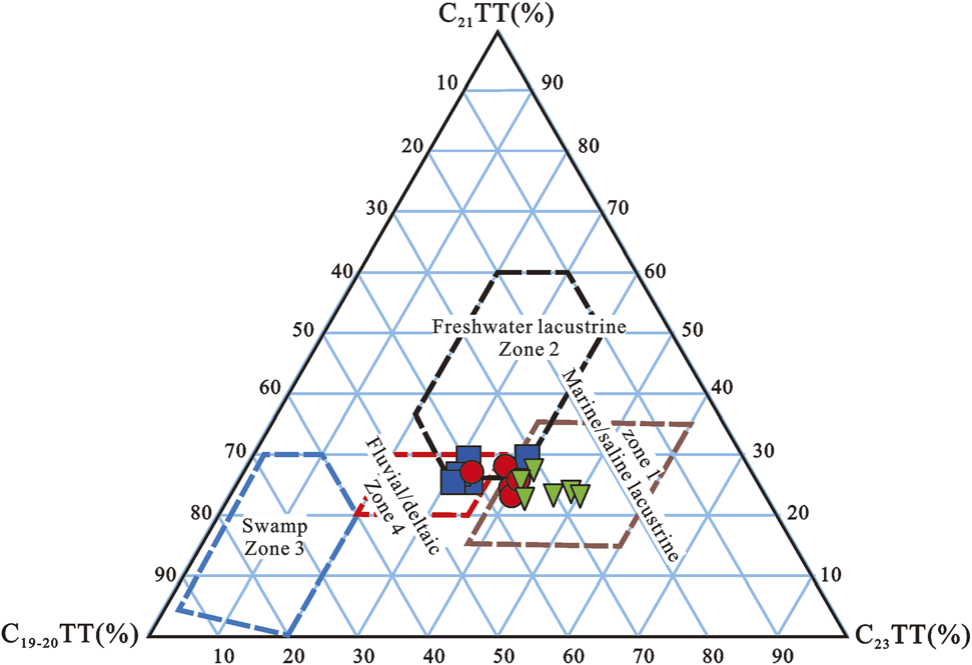
Ternary plots of C_19_TT-C_23_TT (tricyclic terpanes) in source rocks from Offshore Niger Delta Basin, Nigeria.^169,170^

A ternary diagram based on the relative abundance of C_19_–C_23_TTs (C_19+20_TT, C_21_TT, and C_23_TT) (Fig. 12) was successfully used to differentiate the depositional environments of source rocks and crude oils, and the study revealed that four distinct sedimentary environmental zones could be distinguished: marine/saline lacustrine, freshwater lacustrine, fluvial/deltaic, and swamp.^170,173,174^ Based on C_19_–C_23_ TT, Ogbesejana *et al.* (2020) found that rock samples from the Niger Delta Basin received a mixed input of marine and terrigenous organic matter and were deposited under oxic to sub-oxic conditions in lacustrine-fluvial/deltaic sedimentary conditions (Fig. 11).^170^

#### 5.1.4 Hopanes

Hopane (IX) is the name given to a class of aliphatic biomarkers that have a pentacyclic terpenoic structure with a five-membered E-ring. Sediments, hydrocarbons, and coals all contain hopanes in the C_27_ to C_35_ range.^175^ Biomarkers for bacteria and cyanobacteria are considered as hopanoids.^176^ Hopanoids are commonly isotopically light (*δ*^13^C values ranging from −50‰ to −60‰).^177,178^ They are derived from natural precursors such as C_35_-bacteriohopanetetrol (Appendix 1) and similar C_30_- or C_35_-bacteriohopanoids found in the cell membranes of a wide variety of bacteria.^179,180^ These compounds are the counterparts of sterols, which are found in eukaryotes.^179,180^ Hopanes and their biological precursors have been investigated extensively for decades, yet their diagenesis and catagenesis transition processes remain unknown.^181,182^ They are frequently used for both maturity and paleoenvironmental assessments.^6^ For example, high C_35_-/C_31–35_ hopane values (HHI = homohopane index) indicate significantly reduced marine environments.^183^ Bacteriohopanetetrol (Appendix 1) is oxidized to C_32_-hopanoic acid in non-reducing conditions. When the carboxylic group is removed, the fraction of C_30_- and C_31_-hopanes increases. The C_31_/C_30_ hopane ratio (also known as C_31_ 22*R*/C30 hopane) is used to discriminate between marine and lacustrine source-rock depositional settings.^3^ Oils from marine shale, carbonate, and marl source rocks have a higher C_31_ 22*R* homohopane/C_30_ hopane ratio (C_31_*R*/C_30_ > 0.25) than crude oils from lacustrine source rocks. C_30_ hopane, in combination with other parameters such as C_30_*n*-propylcholestane and C_26_/C_25_TT, and the canonical variable from stable carbon isotope analyses, is the best way to differentiate marine from lacustrine crude oils.^3^

In the lacustrine oil samples from the Esprito Santo and Cumuruxatiba basins in Brazil,^114^ 3-methylhopanes and onoceranes were predominant, whereas the relative abundance of 2-methylhopane with an extended side chain was high, and onocerane levels were low only in the marine oil samples from the same basins.^114^ A new index based on methylhopanes was proposed. For oils with a lacustrine origin, the percentage of C_31_ 3β-methylhopane (3MH31) relative to C_30_ hopane (H30), 100 × (3MH31/H30), was >1 while for oils with a marine origin, it was 1.^114^

#### 5.1.5 Gammacerane

Green River Shale extracts were the first to contain gammacerane (X).^184^ The origin of gammacerane, which is widely applied as a salinity indicator, is unknown. The sole known potential biological precursor of gammacerane is tetrahymanoI, a pentacyclic triterpenoid found in protozoa and fungi.^185^ Dehydration of tetrahymanol to create gammacer-2-ene, followed by hydrogenation, is most likely the path of digenetic conversion of tetrahymanol to gammacerane. Sulfurization and subsequent cleavage of tetrahymanol can also produce gammacerane.^186^ Gammacerane is found in a wide range of samples from varied habitats, and its value as a biomarker for salinity is reliant on its relative abundance rather than actual presence.^16^ In marine and non-marine source-rock depositional systems, gammacerane indicates a stratified water column caused by hypersalinity at depth.^186^ In addition to β-carotane and related carotenoids, gammacerane is a prominent biomarker in various lacustrine oils and bitumens, particularly the Green River marl and oils from China.^11,14,135,146,187,188^ Certain marine crude oils derived from carbonate-evaporite source rocks are similarly high in gammacerane.^11,16,187,189,190^ Gammacerane can be used to distinguish between different petroleum families. For example, Poole and Claypool (1984) used gammacerane to distinguish oils and bitumens from different source rocks in the Great Basin.^191^

#### 5.1.6 Steranes

**Fig. 13:**
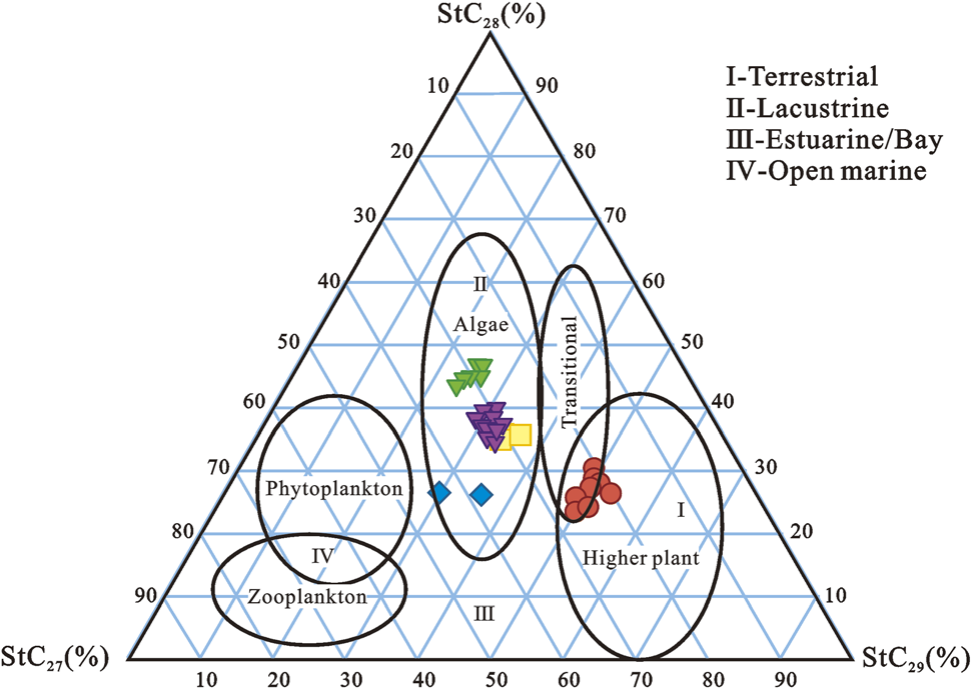
Ternary plot of C_27_, C_28,_ and C_29_ sterane distributions in source rocks from Niger Delta.^18,192^

Steranes (XI) are derived from sterols, which are widely distributed in plants and microorganisms, and are typically found in mature sediments and crude oils *via* diagenesis.^127^ Sterols are classified as biomarkers of animals and zooplankton, algae, or higher plants based on their C-4 or C-24 substitution.^6,192–194^ Steranes are key indicators of source facies, depositional environments, and thermal maturity found in crude oils and sedimentary organic matters.^195^ Sterols are naturally occurring precursors to steranes and diasteranes.^139^ Sterols undergo changes that result in the formation of steranes and diasteranes during diagenesis.^196^ Sterenes and diasterenes are the most likely direct precursors of steranes and diasterenes, respectively.^139^ The hydrogenation produces the saturated isomers. The abundances of C_27_-, C_28_-, and C_29_-steranes and diasteranes provide some insight into the OM origin and depositional conditions (Fig. 13).^7^ C_29_-steranes are thought to be derived mostly from higher plants, whereas C_27_ and C_28_-steranes are thought to originate from phytoplankton.^192^ However, significant amounts of C_29_ steranes have been found in oils and source rocks that are assumed to be mostly marine in origin.^15,197^ Similarly, Grantham (1986) published an important study that showed that the C_29_ steranes in crude oils are not necessarily derived from terrestrial sources.^198^

### 5.2 Aromatic compounds

Aromatic hydrocarbons are important constituents of petroleum and extracts of both recent and ancient sediments,^199–202^ and they have the potential to provide important information on sedimentary environments, source input, migration, thermal maturity, and oil-source rock correlations.^203^ PAHs are not produced by living organisms and are essentially non-existent in natural organic matter.^204^ During diagenesis and catagenesis, the bulk of PAHs in petroleum are the result of complex chemical changes of naphthenic and/or olefinic biological predecessors.^200^ Only in favorable conditions, where a characteristic component of the naphthenic structure has been preserved unchanged, can the biological origin of specific PAHs be determined.^205^ PAHs distributions could be useful in a variety of applications in petroleum geochemistry. Abundance of certain PAHs in sediments and crude oils, such as 1,2,5-trimethylnaphthalene (1,2,5-TMN), 1,2,5,6 tetramethylnaphthalene (1,2,5,6-TeMN), 9-methylphenanthrene (9-MP), 1,7-dimethylphenanthrene (1,7-DMP), originate from diterpenoid and triterpenoid natural products.^200^

#### 5.2.1 Naphthalene

Fossil fuels contain a lot of naphthalene (XII) and its alkylated derivatives.^206,207^ The main precursors for methylated naphthalenes are terpenoids produced from terrestrial plants.^208^ Because of the impacts of source, thermal stress, and biodegradation, their distributions throughout the geosphere are highly varied.^208^ The ratios computed from methylated naphthalenes are thought to reflect an increase in the abundance of the stable isomers relative to the less stable isomers, which is dictated by 1,2-methyl shift and methyl transfer in the naphthalene carbon skeleton.^207^

#### 5.2.2 Phenanthrene

**Fig. 14:**
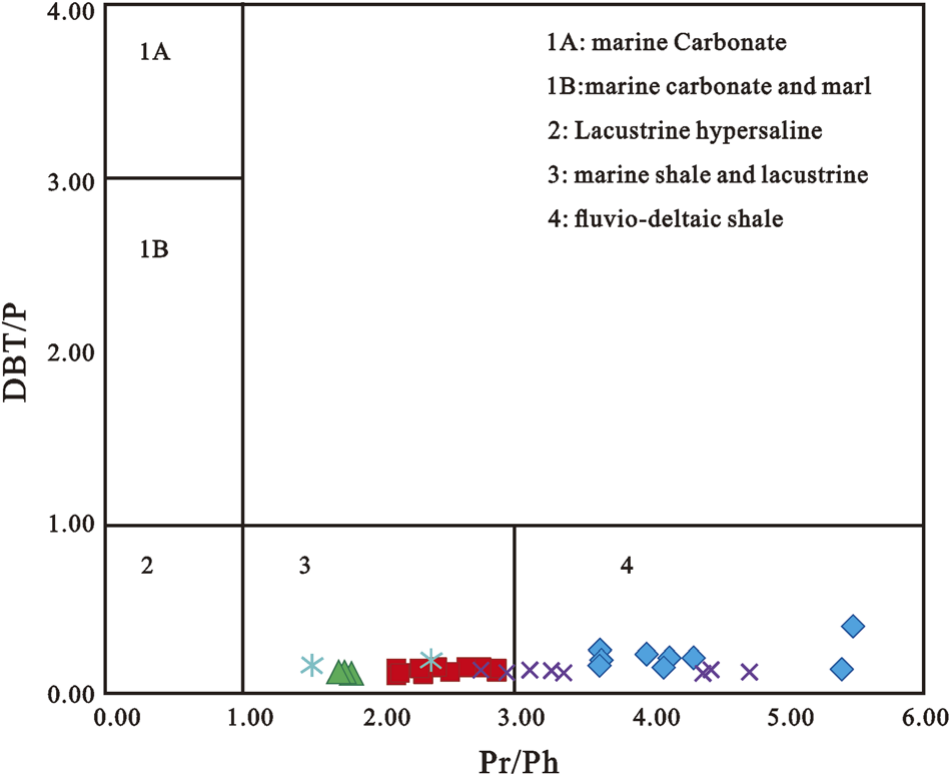
A cross plot of dibenzothiophene/phenanthrene (DBT/P) and pristane/phytane (Pr/Ph) ratios for the Niger Delta oils.^18,212^

In aromatic fractions, phenanthrene (XIII) is a significant component. The relative concentration of phenanthrene in freshwater source rocks was higher than in marine source rocks.^209^ The overall naphthalene/phenanthrene ratio (*∑*_N_/*∑*_P_) of terrestrial freshwater oils was 0.52, that of terrestrial saline oils was 0.64, that of terrestrial hypersaline oils was 0.60, that of marine shales oils was 1.41, and that of marine carbonates oils was up to 1.80. This could indicate that the precursors of phenanthrenes in the crude oils and source rocks investigated come from terrestrial organic matter rather than marine organic matter,^209^ which would be consistent with prior findings.^210,211^ However, Jinggui *et al.* (2005) showed that the composition and abundance of phenanthrene and alkylated analogs in the Tabei and Tazhong uplifts' marine source rocks, as well as the Triassic and Jurassic freshwater source rocks (mudstones and coals) from the Kuche depression, were all very similar.^203^ The findings revealed that, regardless of source, marine and terrestrial organic matter from various sedimentary environments have relatively significant amounts of phenanthrene derivatives, and that the two types of organic matter are not distinguishable. The dibenzothiophene/phenanthrene (DBT/P) ratio alone is an effective predictor of source rock lithology, with ratios >1 for carbonates and ratios <1 for shales.^212^ Hughes *et al.* (1995) and Sivan *et al.* (2008) have successfully applied the cross plots of DBT/P *vs.* PrPh ratios to classify source rock paleodepositional settings (Fig. 14).^208,212^ The classification method is based on the assumption that these ratios indicate diverse Eh–pH regimes arising from substantial microbiological and chemical processes that occur during sediment deposition and early diagenesis. The DBT/P ratio evaluates the availability of reduced sulfur for incorporation into organic matter, whereas the Pr/Ph ratio evaluates the depositional environment's redox conditions.^212^

#### 5.2.3 Triaromatic steroids

**Fig. 15:**
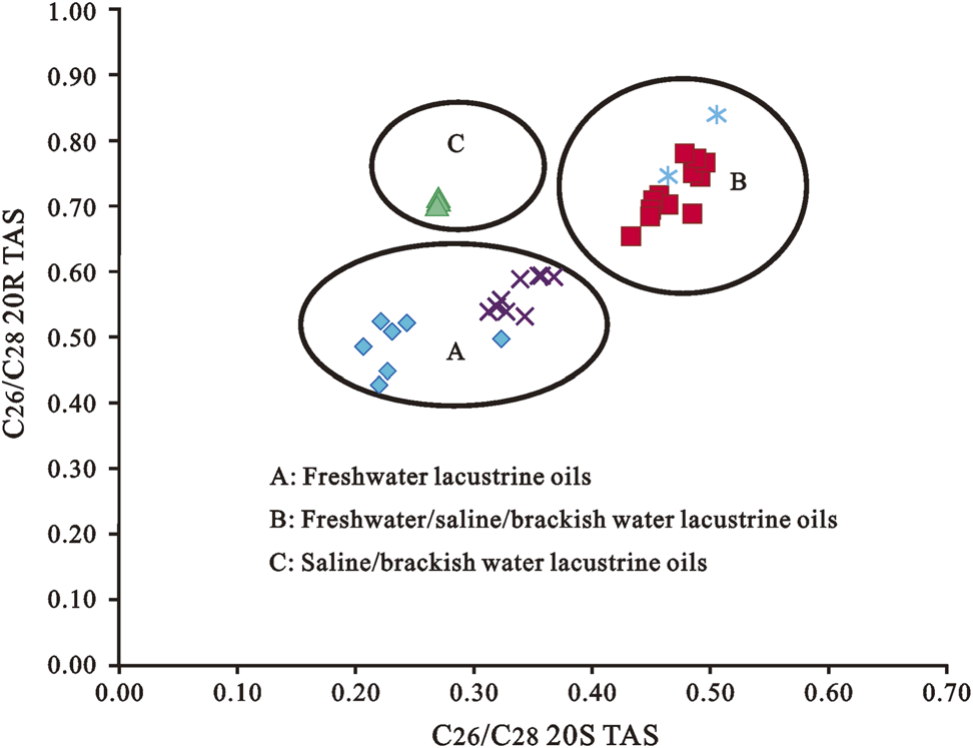
Cross plot of C_27_/C_28_ 20*R* TAS *versus* C_26_/C_28_ 20*S* TAS for Niger Delta crude oils.^215,217^.

Multiple factors determine the relative abundance of triaromatic steroids (TAS) (XIV), which can be utilized as markers for diverse source inputs and depositional settings. C_28_-TAS is commonly found in high abundance in freshwater environments, whereas C_26_-TAS is commonly found in high abundance in saline and brackish water environments.^213–216^ TAS can result from the aromatization of monoaromatic steroids and the loss of a methyl group (–CH_3_).^3^ C_26_, C_27_, and C_28_ triaromatic steroids, like C_27_, C_28_, and C_29_ normal steranes, may retain genetic information about petroleum and source rocks.^3^ Peters *et al.* (2005) and Li *et al.* (2012b) employed a cross plot of C_26_/C_28_ 20*S versus* C_27_/C_28_ 20*R* TAS ratios to identify petroleum systems (Fig. 15).^3,217^ According to Zhang *et al.* (2002) and Mi *et al.* (2007), C_26_ 20*S*, C_26_ 20*R* + C_27_ 20*S*, and C_27_ 20*R* TAS were relatively high in oils derived from Cambrian source rocks, whereas C_28_ 20*S* and C_28_ 20*R* TAS were relatively high in oils derived from Middle-Upper Ordovician carbonate source rocks in the Lunnan oil field, Tarim Basin. TAS has also been used to determine the thermal maturity of crude oils and source rocks.^214,216,218^

#### 5.2.4 Dibenzofurans and benzo[*b*]naphthofurans

**Fig. 16:**
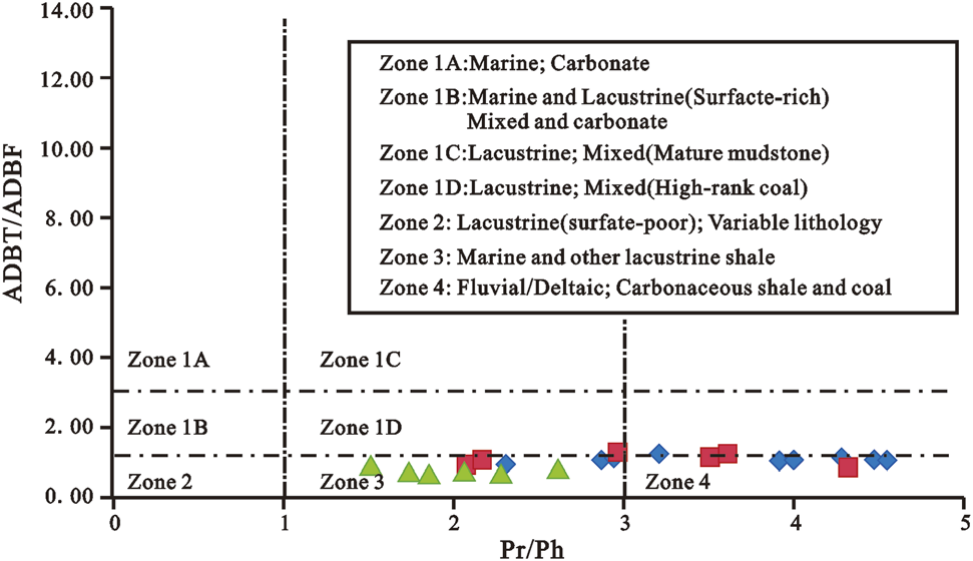
Cross plots of ADBT/ADBF *versus* Pr/Ph.^202^

Dibenzofurans (DBFs) (XV), their alkylated homologs, and benzo[*b*]naphthofurans (BNFs) (XVI to XVIII) are the major oxygen heterocyclic aromatic compounds found in oils, coals, sediment extracts, and tar deposits. The source of DBFs in oils and sedimentary organic matter is still a point of contention. DBFs may be derived from dehydrated and condensed polysaccharides or the oxidative coupling of phenols, according to previous research.^219–221^ DBF, DBT, and biphenyl abundances in Permian rocks (East Greenland) were most likely produced from phenolic compounds in the lignin of woody plants.^222^ The bulk of natural compounds linked to dibenzofuran are lichen or higher fungus metabolites. The carbon–carbon oxidative coupling of orsellinic acid and its homologs appears to be the source of lichen dibenzofurans.^223^ As a result, Radke *et al.* (2000) proposed that DBFs in crude oils and sediment extracts may be used as lichen biomarkers.^202^ However, more research is needed to confirm the lichen-DBF precursor-product link.^224^ Biphenyl with oxygen can produce dibenzofuran, according to simulation experiments and geological evidence.^205^ Similarly, methyl-substituted biphenyls can combine with methylated DBFs to synthesize methylated DBFs. In the laboratory, biphenyl derivatives are often employed as reactants to synthesize dibenzofurans.^223^ To completely comprehend the origin and evolution of DBFs in the geosphere, further geological evidence and laboratory studies are required. Dibenzofuran and its alkylated homologs (abbreviated as DBFs) as well as benzo[*b*]naphthofurans have been used as key molecular markers in organic geochemistry. DBF occurrence and distribution are primarily influenced by source rock type and/or depositional environment.^170,202,209,225^ DBFs appear to predominate in freshwater source rocks, terrestrial oils, and coals,^170,209,225–227^ whereas their sulfur-heterocyclic counterparts dibenzothiophenes (DBTs) appear to predominate in marine shales and carbonates.^212,227,228^ Radke *et al.* (2000), Asif (2010), and Kruge (2000) indicated that the relative abundance of alkyldibenzothiophene (ADBT) compared to alkyldibenzofuran (ADBF) may be used to identify depositional settings (Fig. 16).^202,205,229^ Variations in the relative abundances of fluorenes, dibenzofurans, and dibenzothiophenes have been reported to be a reliable indicator of source rock sedimentary settings.^203,230^ The concentrations of the dibenzothiophene series were high in marine carbonate source rocks, while those of fluorenes and dibenzofurans series were high in freshwater source rocks.^209,226,227,230^ As a result, the fluorene, dibenzofuran, and dibenzothiophene series can be further used in the oil-source rock correlation study.

Li and Ellis (2015) identified benzo[*b*]naphthofurans (benzo[*b*]naphtho[2,1-*d*]furan (BN21F), benzo[*b*]naphtho[1,2-*d*]furan (BN12F), and benzo[*b*]naphtho[2,3-*d*]furan (BN23F)) in crude oils and rock extracts by co-injecting authentic standards and proposed that this ratio may be a potential molecular geochemical parameter to indicate oil migration pathways and distances.^224^ In the Northern (Poland) and Southern (Argentina), these compounds have been found in bitumen from fluvial-deltaic siltstone and charcoal from Jurassic records of wildfires.^231,232^ There is currently no clear understanding of the factors that influence benzo[*b*]naphtho[*d*]furan isomerization, and the source of these compounds is unknown. Such oxygenated chemicals, on the other hand, are thought to come from terrigenous organic matter, which would explain their high abundance in coal and coaly shales.^224^ Li and Ellis (2015) found BNFs in fluids and source rocks from various depositional settings in a more extensive investigation of the BNFs.^224^ In pyrolysates from a sub-bituminous coal (random vitrinite reflectance Rr = 0.42 percent) and a high volatile bituminous coal (random vitrinite reflectance Rr = 0.56%), Vukovic *et al.* (2016) found a larger abundance of [2,1] and [1,2]BNFs compared to [2,3]BNF. They suggested that the water produced by kerogen, in combination with the clay minerals, undergoes numerous interactions with OM, and that these reactions could be the source of diverse oxygenated PAHs.^233^ Cesar and Grice (2017), recently reported benzonaphthofurans in crude oils and source rocks from the Dampier sub-basin in Western Australia, noting that clay catalysis appears to impact the formation of [1,2]BNF and that the ratio [2,1]/[1,2]BNF might be utilized to explain lithofacies. When compared to clay-depleted sediments from marine environments, this ratio was substantially lower in the fluvial-deltaic system (carbonate sequences). The authors proposed that the ternary plot of [2,1]-[1,2]-[2,3]BNFs could be used for fluid–fluid and fluid–source rock correlations based on their findings.^234^

## 6 Conclusion and outlook

This paper reviews the early and modern applications of biomarkers for paleoenvironmental reconstruction. The oxic and anoxic depositional conditions, and lacustrine and marine depositional environments were compared. The experimental and instrumental methods for analyzing biomarkers in shales were then discussed. Saturated and aromatic biomarkers were discussed as indicators of marine sedimentary depositional environments, fluvial/deltaic, freshwater, saline/brackish water depositional environments, and redox conditions. This review showed that biomarkers could be used to establish the sedimentary depositional environments, redox conditions, and organic matter enrichments of shales which are critical to deep energy exploitation. However, because biomarker geochemistry is a rich field for the paleoenvironmental reconstruction of source rocks and oils, many biomarker classes remain to be discovered and studied for the understanding of paleoenvironmental reconstruction. Hence, abundant opportunities exist for exploring new classes of biomarkers and their paleoenvironmental significance. Improvements can be made in the chromatographic separation and instrumental analyses of biomarkers by applying some automatic instruments that can separate EOM and oils to SARA and GC × GC-TOFMS which can eliminate the problems of co-elution and poor resolution usually encountered in GC-MS and at times GC-MS/MS. Also, for a more reliable paleoenvironmental reconstructions, there is a need to combine isotopic, elemental and maceral proxies with biomarkers.

## Appendix 1: biomarkers mentioned in the text

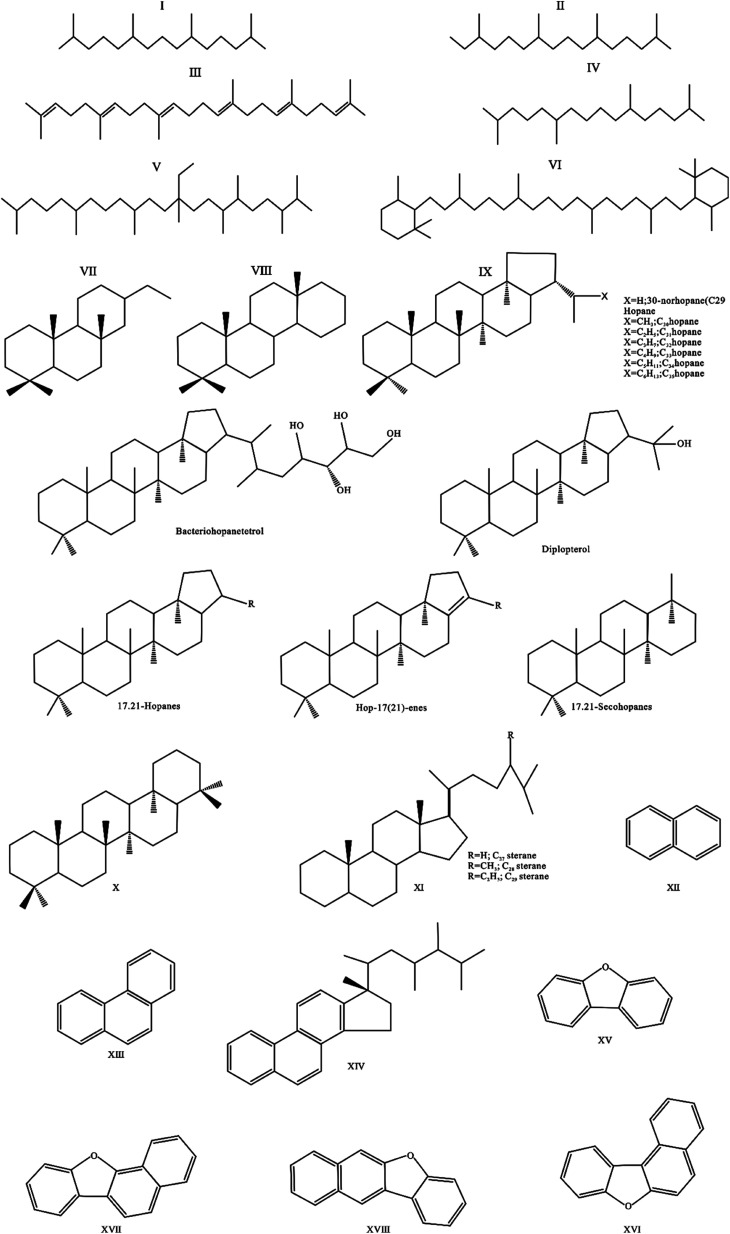



## Author contributions

Conceptualization, Abiodun Busuyi Ogbesejana and Bo Liu; writing—original draft preparation, Abiodun Busuyi Ogbesejana; writing—review and editing, Abiodun Busuyi Ogbesejana, Bo Liu, Oluwasesan Michael Bello, Segun Ajayi Akinyemi, Yu Song and Shuo Gao; supervision, Bo Liu; project administration, Abiodun Busuyi Ogbesejana and Bo Liu; funding acquisition, Bo Liu. All authors have read and agreed to the published version of the manuscript.

## Conflicts of interest

The authors declare that there is no conflict of interest regarding the publication of this paper.

## Supplementary Material
